# Dynamics of Cell Shape Inheritance in Fission Yeast

**DOI:** 10.1371/journal.pone.0106959

**Published:** 2014-09-11

**Authors:** Juan F. Abenza, Anatole Chessel, William G. Raynaud, Rafael E. Carazo-Salas

**Affiliations:** The Gurdon Institute, University of Cambridge, Cambridge, United Kingdom; Cancer Research UK London Research Institute, United Kingdom

## Abstract

Every cell has a characteristic shape key to its fate and function. That shape is not only the product of genetic design and of the physical and biochemical environment, but it is also subject to inheritance. However, the nature and contribution of cell shape inheritance to morphogenetic control is mostly ignored. Here, we investigate morphogenetic inheritance in the cylindrically-shaped fission yeast *Schizosaccharomyces pombe*. Focusing on sixteen different ‘curved’ mutants - a class of mutants which often fail to grow axially straight – we quantitatively characterize their dynamics of cell shape inheritance throughout generations. We show that mutants of similar machineries display similar dynamics of cell shape inheritance, and exploit this feature to show that persistent axial cell growth in *S. pombe* is secured by multiple, separable molecular pathways. Finally, we find that one of those pathways corresponds to the *swc2-swr1*-*vps71* SWR1/SRCAP chromatin remodelling complex, which acts additively to the known *mal3-tip1-mto1-mto2* microtubule and *tea1-tea2-tea4*-*pom1* polarity machineries.

## Introduction

Every cell has a characteristic shape, which is fundamental to its fate and function [1], [2]. Cell shape is not only the product of genetic design and of the physical and biochemical environment but it is also subject to inheritance, as every cell stems from a previous cellular entity with a given composition and morphology [3], [4]. In the last two decades many studies have demonstrated the importance that transmission from cells to their progeny of molecules – DNA, proteins or even prions - and intracellular organelles – including nuclei - can have in the development and fate of cells, tissues and organisms. However, the fact that general morphological properties may also be inheritable and impact on the shape and growth pattern of a cell‘s progeny has seldom been addressed.

Here, we investigate morphogenetic inheritance in cells of the unicellular fission yeast *Schizosaccharomyces pombe*. Fission yeast cells are cylindrically-shaped, walled cells which grow axially from their exactly opposed cell ends - by remodelling the cell wall exclusively at cell ends - in a way that tightly regulates cell shape within and across generations. Following mitosis, during which cells do not grow, wild-type *S. pombe* cells re-initiate growth first monopolarly from their ‘old end’ (inherited from their mother cell) and later during interphase initiate bipolar growth by activating their ‘new end’ (derived from the site of cell division), in an event termed ‘new end take off’ or NETO [5], [6]. After cells double their original size, bipolar growth stops and cells re-enter mitosis where they divide by medial fission, giving rise to two genetically identical and equally-sized daughters. This pattern of polarized growth, which contributes to the perpetuation of a uniform cell geometry and to correct volumetric and chromosomal equipartition during mitosis, is regulated by an intracellular network of gene and protein machineries that include microtubules, actin, polarity factors, trafficking factors and regulators of cell wall synthesis [7]–[10]. Correspondingly, disruption of those machineries either by gene mutation or deletion (gene knock-out) can lead to a discrete variety of cell shape and growth pattern defects including monopolar, wide, orb (round), T-shaped, skittle (drop-shaped) and bent/curved (non-axially straight) [1], [2], [11]–[13].

Focusing on the shape regulation and inheritance of ‘curved’ mutants, which fail to grow axially straight, we quantitatively characterize their cell shape throughout generations and show that mutants of similar machineries display similar dynamics of cell shape inheritance. Based on those patterns, we find evidence that there are multiple, separable pathways that secure persistent axial cell growth in this species, suggesting the existence of convergent molecular cascades controlling that specific aspect of morphogenesis.

## Results

### Cell shape is inherited across cell generations following defined dynamics

In order to investigate morphogenetic inheritance in *S. pombe* we decided to focus on ‘curved’/‘bent’ mutants, in which the direction of growth has been shown to frequently deviate from straight. Of the ∼3700 non-essential genes of *S. pombe*, we identified and chose 16 genes whose deletion causes non-straight axial growth phenotypes (Table 1 and [3], [4], [11]). They included genes with a known role in cell polarity or cytoskeleton like *tea1* (a Kelch-repeat containing polarity factor), *tea2* (a kinesin), *tea4* (a Tea1 interactor), *pom1* (DYRK kinase) and *mto1* (centrosomin-related microtubule nucleator), but also genes not related to those machineries like *vps71* and *swr1* (chromatin remodelling), *rpl3702* (ribosomal structure), and *mss116* and *mgr2* (predicted mitochondrial; www.pombase.org).

**Table 1:** Overall penetrance of the curved phenotype in each of the strains listed.

Deletion	Function of the proteinwhose gene is deleted	Overallpenetrance
*alp14Δ*	Microtubule cytoskeleton organization	38.2%
*mal3Δ*	Microtubule associated protein	39.5%
*mgr2Δ*	Mitochondrial membrane protein(inferred)	13.5%
*mss116Δ*	Mitochondrial RNA-helicase (inferred)	16.3%
*mto1Δ*	Microtubule nucleation	31.9%
*mto2Δ*	Microtubule nucleation	49.7%
*pom1Δ*	Cell morphogenesis	27.2%
*ria1Δ*	Ribosome biogenesis (inferred)	15.3%
*rpl3702Δ*	Ribosomal protein 60S (inferred)	17.9%
*swc2Δ*	Chromatin remodelling,regulation of transcription	32.6%
*swr1Δ*	SNF2 Helicase, chromatinremodelling	28.8%
*tea1Δ*	Polarity establishmentand maintenance	41.7%
*tea2Δ*	Kinesin, microtubule motor	40.2%
*tea4Δ*	Polarity establishment andmaintenance	37.3%
*tip1Δ*	Microtubule cytoskeleton organization	36.8%
*vps71Δ*	Chromatin remodelling (inferred)	30.2%
wild-type		7.6%

The number of cells measured varied depending on the mutant, from 173 to 497. The function of the protein encoded by the gene deleted in each case, described in the bibliography or inferred in silico, is shown in the middle column.

**Figure 1:**
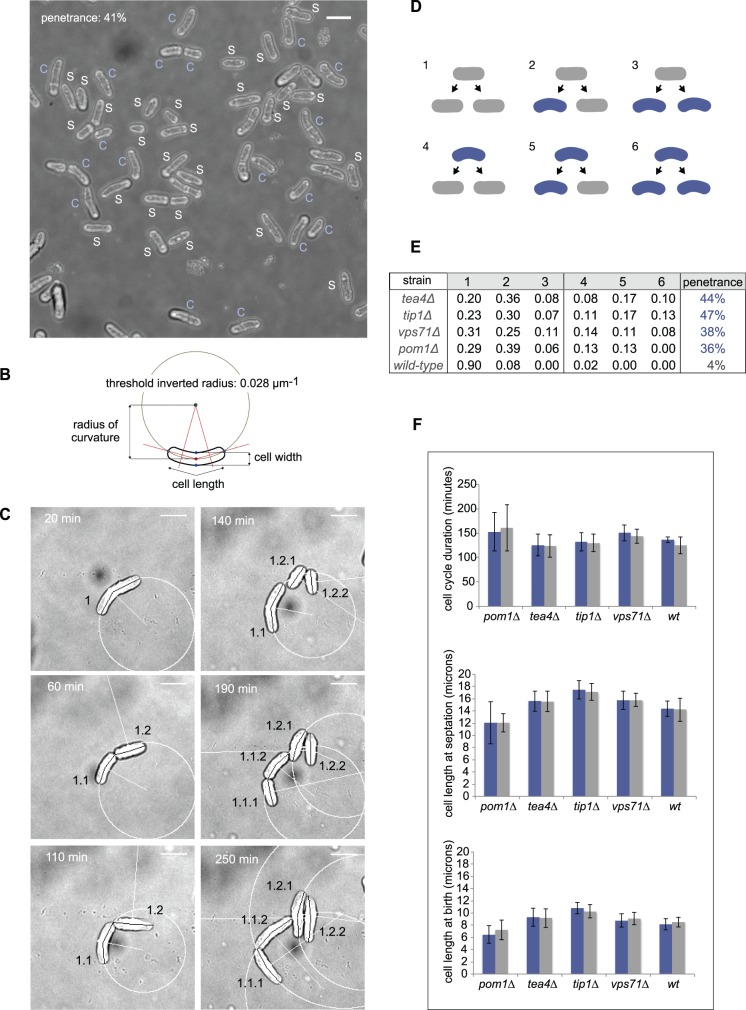
Dynamics of cell shape inheritance of curved and straight cells from genotypically identical *S. pombe* populations. **A**. Visual classification of curved (C) and straight (S) cells in the ‘curved’ mutant *tea4Δ*. The overall penetrance percentage was calculated by dividing the number of cells visually classified as curved (18) by the total number of cells (44). Bar, 10 µm. **B**. Quantitative classification of curved and straight cells. The radius of curvature of each cell was quantitatively estimated by calculating the inverted radius of a circle crossing the cell centre and two ends. Cells with an inverted radius of curvature greater than radius^−1^ = 0.028 µm^−1^ were considered curved. **C**. Time-lapse image sequence showing the lineage of a single *tip1Δ* cell over two rounds of cell division. Images were taken every 10 minutes and curvature was measured for each cell after birth and before septation. Unique name identifiers were given to each daughter and grand-daughter cell to indicate its origins, e.g. the daughters of 1 are 1.1 and 1.2. For cells selected as ‘curved’, a circle of radius equal to the cell’s radius of curvature is drawn around it. See also Electronic Movie S1. Bar, 10 µm. **D**. The six types of morphological cell division outcomes observed in a mixed population containing curved and straight cells. **E**. The frequencies of those six outcomes over the entire cell lineage were calculated for four curved mutants and the wild-type, based on the phenotype of progenitors and progeny before cell septation. The penetrance at septation for each strain is shown. **F**. Duration of cell cycle and cell length, at birth and before septation, of curved (blue) and straight (grey) cells belonging to each of the indicated strains.

When grown exponentially in suspension, mutants in each of those genes gave rise to a mixture of visibly straight and non-straight cells with different degrees of phenotypic aberration (Fig. 1A and Fig. S1). Interestingly, close inspection revealed that, contrary to what is observed during growth out of stationary phase or at restrictive temperatures [5], [6], [10]–[12], in exponentially growing mutants bent cells were quite rare (in our definition, bent cells are those displaying a sharp angular deviation of >15 degrees between two major cell segments), accounting for only 9.23% of all non-straight cells observed in *alp14Δ*, 11.94% in *pom1Δ*, 9.59% in *tea1Δ*, and less than 7% in all other mutants analysed (Fig. S1 and Table S2). Given the relative rarity of the bent phenotype and, conversely, the predominantly curved (smoothly-deviating, arc-like) phenotypic defect of the mutants, we are referring to the phenotypic defect of all of them as ‘curved’ from this point onwards for simplicity.

In order to quantitate the mutants‘ phenotypes objectively and reproducibly, we imaged cell populations for each of them and used semi-automated image analysis to estimate the inverse radius of curvature (radius^−1^) of each cell for every mutant analyzed (see Materials and Methods). We then visually inspected a few populations of cells, decided which cells in those populations looked straight and which looked curved, and based on this visual decision we identified the radius^−1^ = 0.028 µm^−1^ as a quantitative threshold that allows distinguishing straight from curved cells (Fig. 1B and Materials and Methods).

When quantitated in this way, despite their overall superficial phenotypic similarity, different mutant cell populations were found to display different percentages of curved cells (‘penetrances’; Table 1), which varied from ∼13% (in the wild-type ∼8% of the cells have a radius below the threshold) to ∼50% (for example *mto2Δ*). Penetrances were approximately constant across biological repeats under the same culture conditions (not shown), correlated only mildly with the degree of curvature of the mutants (Fig. S2) and did not correlate with other geometric variables such as cell length (which differs in each mutant) and width (which is mostly constant).

To clarify how different penetrances arise, we decided to image with time-lapse microscopy 20–40 cells for four of the mutants - *tip1Δ*, *tea4Δ*, *pom1Δ* and *vps71Δ* - as they grew and divided through four generations under the microscope, to quantitate the frequency of appearance of straight and curved cells in their progeny. Conspicuously, both straight and curved cells were able to generate curved and straight cells after cell division (Fig. 1C, Fig. S3, Table S3 and Movies S1–S4 and S6; note that we refer, unless specified, to ‘adult’ mother and daughter cells, i.e. cells in their final stage before they septate). Quantitation of the frequencies of the six types of cell division outcome observed - straight parent to straight-straight, straight-curved or curved-curved daughters, or curved parent to straight-straight, straight-curved or curved-curved daughters - revealed that the mutants have different frequencies, suggesting different dynamics of cell shape inheritance (Figs. 1D–E). Importantly, the penetrances did not result from differential proficiency of straight and curved cells in the different mutants, as neither cell length (after division or before septation) nor cell cycle duration differed between both phenotypes of cells, for any of the four mutants (Fig. 1F).

Quantitative analysis of cell shape inheritance in all other mutants (see Movie S5 for an example) confirmed these observations and indicated that knockouts of similar machineries may have similar inheritance frequencies, suggesting that a given steady-state penetrance might be generated by common inheritance rules (Table 2). However the inverse was not true. For example, *swc2Δ* and *swr1Δ* had very similar penetrances and displayed very similar frequencies for the different cell division outcomes but *mto1Δ*, which showed an almost identical penetrance, displayed very different frequencies (Table 2). Thus, cell shape is inherited across generations following dynamical rules that vary depending on the cellular genotype.

**Table 2:** Frequency (that is, proportion of incidences) of each of the six types of cell shape division outcome over the entire cell lineages analysed for the sixteen curved mutants and the wild-type.

Deletion	1	2	3	4	5	6	Measuredpenetrance	Predictedpenetrance
*alp14Δ*	0.59	0.22	0.00	0.12	0.07	0.00	34%	15%
*mal3Δ*	0.27	0.27	0.08	0.12	0.19	0.06	37%	38%
*mgr2Δ*	0.74	0.17	0.02	0.06	0.01	0.01	11%	11%
*mss116Δ*	0.58	0.26	0.02	0.10	0.04	0.00	12%	17%
*mto1Δ*	0.26	0.34	0.05	0.18	0.15	0.03	35%	32%
*mto2Δ*	0.12	0.27	0.12	0.10	0.25	0.14	57%	52%
*pom1Δ*	0.29	0.36	0.08	0.10	0.13	0.05	20%	37%
*ria1Δ*	0.56	0.26	0.02	0.10	0.06	0.00	18%	18%
*rpl3702Δ*	0.59	0.21	0.03	0.11	0.05	0.00	12%	17%
*swc2Δ*	0.45	0.24	0.05	0.06	0.13	0.07	28%	31%
*swr1Δ*	0.41	0.29	0.03	0.07	0.17	0.03	30%	29%
*tea1Δ*	0.24	0.28	0.10	0.12	0.20	0.08	43%	41%
*tea2Δ*	0.22	0.24	0.12	0.12	0.19	0.11	44%	45%
*tea4Δ*	0.20	0.26	0.11	0.11	0.16	0.16	50%	48%
*tip1Δ*	0.23	0.30	0.07	0.11	0.17	0.13	48%	43%
*vps71Δ*	0.26	0.27	0.07	0.11	0.21	0.07	42%	38%
wild-type	0.90	0.08	0.00	0.02	0.00	0.00	5%	4%

Measurements are based on the phenotype of progenitors and progeny before cell septation. 1–6 are represented in Fig. 1D. The columns on the right indicate the measured penetrance at septation (left) and predicted penetrance at septation (by the Markovian model) based on the six frequencies (right), for each mutant. Importantly, the measured penetrances and the frequencies come from independent biological experiments. For all strains other than *alp14Δ* and *pom1Δ* both penetrances were within ±5% difference. This likely due to the fact that *alp14Δ* cells often do not undergo division (not shown) and that *pom1Δ* cells divide highly asymmetrically [18], both features which are not accounted for by our model.

### Cell shape is actively modulated by multiple factors that impact on growth pattern throughout generations

**Figure 2:**
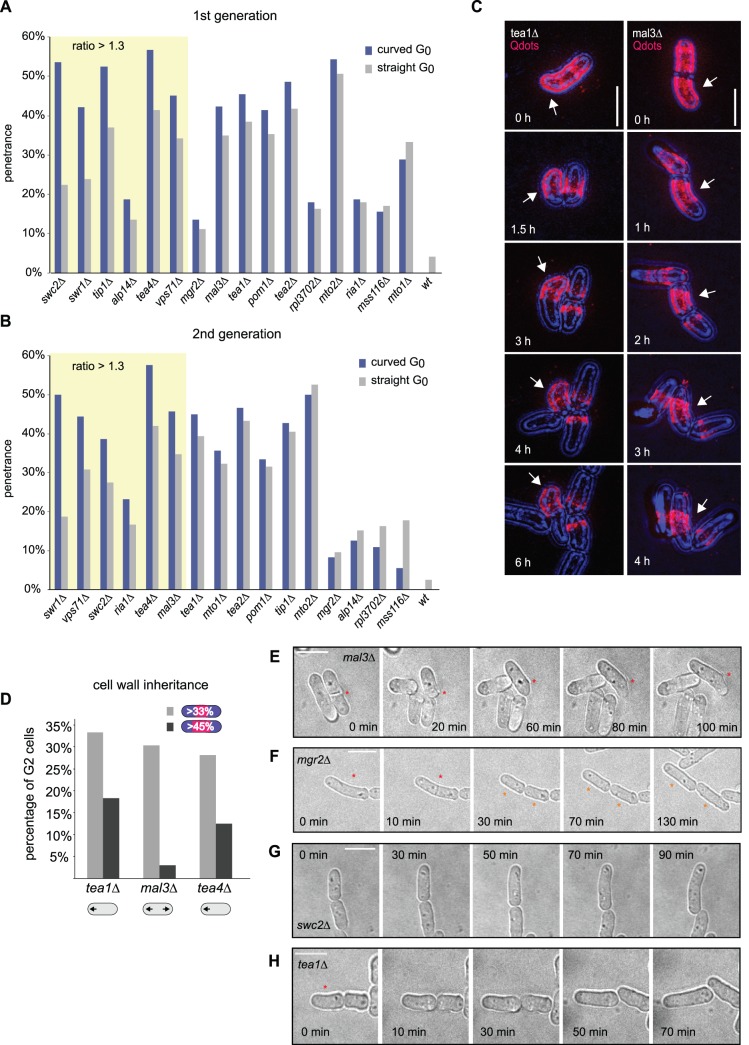
Active modulation of cell shape throughout generations. **A**. Percentage of septating curved cells resulting from divisions of curved (blue) and straight (grey) mothers, for each mutant and the wild-type. Strains exhibiting a ratio between the first and the second percentages higher than 1.3 are highlighted by a yellow box. **B**. Percentage of septating curved cells resulting from curved (blue) and straight (grey) grandmothers. Strains exhibiting a ratio between the first and the second percentages higher than 1.3 are highlighted by a yellow box. **C**. Image sequences showing the inheritance, during two cell cycle rounds, of the Qdot-tagged cell wall (see Materials and Methods) of a *tea1Δ* and a *mal3Δ* cell lineage. Images are Z-stack maximal intensity projections of the medial 2 µm of cells. In both cases, curved cell wall segments (arrows) are transmitted practically unaltered from mother cells to their progeny, influencing not only their initial but also their final morphology. Note that the bottom *tea1Δ* cell shown rotated slightly. Such rotations were exceptional and rotating cells were not included in the quantitative analysis. **D**. Percentage of granddaughter cells (G2) that retain more than a third (light grey) or than 45% (dark grey) of the cell wall of their grandmother, for two monopolar mutants (*tea1Δ* and *tea4Δ*) and the bipolarly growing mutant *mal3Δ*. **E**. A *mal3Δ* cell that divides asymmetrically (red asterisk) predisposing one of its daughters to grow curved from the newly generated cell end. **F**. A ‘curved’ *mgr2Δ* cell (red asterisk) with two seemingly straight segments joined at an angle of 22**°** that, after division, produces two straight descendants (orange asterisks). **G**. Straight-to-curved (StoC) transition of a growing *swc2Δ* cell. **H**. Curved-to-straight (CtoS) transition of an initially curved *tea1Δ* cell (red asterisk) that ‘corrects’ its shape dynamically by growing. All bars represent 10 µm.

Given that both straight and curved cells were apparently able to generate curved and/or straight cells after cell division, we asked whether for a given mutant (i.e. for a given genotype) the phenotypic outcome of a cell is influenced or not by the phenotype of its mother. We therefore quantitated the phenotypic inheritance patterns of curved and straight cells separately. Notably, we found that straight cells frequently generate at least one straight daughter and that curved cells tend to give rise to at least one curved daughter, apparently regardless of the penetrance of the mutant examined (Table 2). This effect became particularly obvious for example when we quantitated the percentage of curved cells generated from straight cells and compared it with that generated from curved cells, and found the first percentage to be often much lower than the second (Fig. 2A). It was similarly apparent for many mutants when we calculated the percentage of curved cells originating from straight cells versus that originating from curved cells after two generations (that is, the percentage of curved ‘grandchildren’ stemming from each type of cell; Fig. 2B).

To rule out that this effect was due to the cells growing and dividing transiently out of steady-state (for example, due to the fact they were constrained suddenly to grow on glass-bottom imaging plates rather than in suspension), we tested whether the six frequencies of phenotypic inheritance we calculated using measurements from four cell generations reflected unequivocally the inheritance pattern of the different mutants. For this purpose we developed a mathematical first order Markovian model. A first order Markovian model of a process is a stochastic model where the state of a system depends probabilistically only on its immediately preceding state. In our case, the model described, for each mutant and the wild-type, the evolution in their number of curved and straight cells as a function of time. In the model, we assumed that the number of curved and straight cells in a population at a given generation depended only on: a) the number of curved and straight cells in the previous generation, and b) the six cell division outcomes possible for each dividing cell, with the probabilities of the six outcomes equal to the six experimentally measured inheritance frequencies. Based on those assumptions, we used the model to calculate the predicted proportion of curved cells in the population (i.e. the ‘predicted penetrance’) when the population would reach a steady state (see Materials and Methods). As shown in Table 2, we found that for all strains except for *alp14Δ* and *pom1Δ* the predicted and measured penetrances were within ±5% difference of each other (*alp14Δ* cells often do not undergo division and *pom1Δ* cells divide highly asymmetrically [7]–[10], [14], both features unaccounted for by the model). Given the very few assumptions made by the first order Markovian model, this suggests that to a first approximation the measured frequencies of phenotypic inheritance are unequivocal qualifiers of the shape inheritance pattern and suffice to give rise to the shape inheritance patterns and overall penetrances we see experimentally (that is, the rates and the penetrance are univocally coupled). Importantly it indicates that our experimental conditions and the data acquired are reflective of a steady-state growth pattern.

We asked next what factors could contribute to a cell’s shape having an impact on the cell shape and growth pattern of its progeny after one or two generations. We reasoned that one such factor could be the cell wall, which is inherited at cell division and whose properties rely on the cell expansion pattern. To investigate this possibility, we decorated the cell wall of three mutants (*tea4Δ*, *tea1Δ* and *mal3Δ*) with fluorescent quantum dots (Qdots; see Materials and Methods), which allowed us to study cell wall inheritance in ‘live’ cells during three generations by looking at the geometry of the labelled cellular fragments inherited. We observed that relatively big straight and curved Qdot-tagged cell wall fragments remained intact for at least two generations, conditioning the geometry of a portion of the progeny (Fig. 2C–D; 28% of G2– second generation - *tea4Δ* cells, 33% of G2 *tea1Δ* cells and 30% of G2 *mal3Δ* cells contained at least one third of the Qdot-tagged G0 cell wall of their grandmothers; n = 60, 33 and 64, respectively). Interestingly, when we measured the percentage of cells that inherited at least 45% of the grandmother’s cell wall, we found that it was significantly higher in monopolarly growing mutants (18% for *tea1Δ* and 13% for *tea4Δ*; Fig. 2D) than in bipolarly growing mutants (3% for *mal3Δ*; Fig. 2D). This indicates that the cell wall contributes to keeping a transient ‘template’ of previous cellular growth pattern, in a way that differs for different genotypes.

We also asked whether other factors, such as the way in which cell division occurs (which likely varies from genotype to genotype) influences daughter cell shape. Therefore we measured for each mutant the frequencies of the six cell division outcomes possible before and after cytokinesis (i.e. by measuring the curvature of the mother cells immediately before septation and of daughters immediately after). We observed that straight cells frequently produce at least one curved cell immediately when they divide and vice versa (Table 3). This was the case even for wild-type cells, where one out of four straight cells generated at least one curved descendant immediately after cell division. One reason for this high frequency of curved daughter generation could be abnormal septation (Fig. 2E), which could directly modify or predispose daughter cells to acquire or lose the curvature they would have adopted otherwise. This could be generally a problem in mutants initiating growth primarily from the septum-derived cell end, after cell division. This is likely the case in *pom1Δ*, where 23.2% of cells display an oblique septum and where around 30% of the cells fail to resume growth from the previously growing end [15]. However, we found that in other mutants such a cytokinetic defect is very infrequent (Table S2). This indicates that other factors might be at play (for example, Fig. 2F shows a case in which two ‘straight’ cells were generated immediately after the division of an overall ‘curved’ cell that, prior to division, seemed to be composed of two adjoined straight segments).

**Table 3:** Frequency (proportion of incidences) of each of the six types of cell shape division outcome over the entire cell lineages analysed for the sixteen curved mutants and the wild-type.

Deletion	1	2	3	4	5	6
*alp14Δ*	0.52	0.43	0.06	0.53	0.35	0.12
*mal3Δ*	0.51	0.39	0.10	0.34	0.47	0.18
*mgr2Δ*	0.68	0.30	0.02	0.58	0.33	0.08
*mss116Δ*	0.64	0.31	0.05	0.56	0.31	0.12
*mto1Δ*	0.41	0.51	0.08	0.33	0.41	0.26
*mto2Δ*	0.67	0.27	0.06	0.34	0.49	0.18
*pom1Δ*	0.54	0.40	0.06	0.50	0.40	0.10
*ria1Δ*	0.66	0.33	0.01	0.60	0.30	0.10
*rpl3702Δ*	0.66	0.32	0.02	0.50	0.35	0.15
*swc2Δ*	0.69	0.22	0.09	0.43	0.46	0.11
*swr1Δ*	0.73	0.23	0.03	0.54	0.33	0.13
*tea1Δ*	0.51	0.36	0.12	0.35	0.50	0.15
*tea2Δ*	0.43	0.45	0.12	0.25	0.54	0.21
*tea4Δ*	0.53	0.41	0.07	0.33	0.48	0.19
*tip1Δ*	0.37	0.41	0.22	0.21	0.53	0.26
*vps71Δ*	0.76	0.16	0.08	0.50	0.34	0.16
wild-type	0.73	0.24	0.02	0.33	0.67	0.00

Measurements are based on the phenotype of progenitors before cell septation and progeny immediately after cell septation at birth. 1–6 are represented in Fig. 1D. The frequencies are separated and normalized in two groups based on the progenitor’s shape: 1–3, straight progenitor; 4–6, curved progenitor.

Finally, we quantitated the contribution of active changes in growth pattern - between the time a cell is born (the second recorded timepoint after cytokinesis) and the time it divides - in determining cell shape fate, for all genotypes/mutants (Table 4). We found that for a given mutant the frequency at which cells change from straight to curved (StoC) is related to its penetrance, that is, that straight cells belonging to strains with higher penetrance are more likely to spontaneously modify their direction of growth and become curved (see Fig. 2G for an example). We also found that the frequency of changing from curved to straight (CtoS; see Fig. 2H for an example) was, with the exception of *mto2Δ* and *tea4Δ*, higher than that of going from StoC. This indicates that genotypically *S. pombe* has a default tendency to grow straight. Interestingly, although the CtoS frequency was also generally higher in mutants with higher penetrances, its correlation with the StoC frequency was not straightforward. In addition, we found that there was no obvious predictive correlation between the instantaneous local curvature of a cell and how its end growth geometry evolves and/or is inherited (Figure S4).

**Table 4:** Frequency with which straight and curved cells change or maintain their shape during growth, considering exclusively their initial and final geometry (after birth and before septation).

Deletion	1	2	3	4
*alp14Δ*	0.90	0.10	0.71	0.29
*mal3Δ*	0.72	0.28	0.43	0.57
*mgr2Δ*	0.90	0.10	0.74	0.26
*mss116Δ*	0.85	0.15	0.78	0.22
*mto1Δ*	0.70	0.30	0.66	0.34
*mto2Δ*	0.53	0.47	0.34	0.66
*pom1Δ*	0.68	0.32	0.52	0.48
*ria1Δ*	0.80	0.20	0.90	0.10
*rpl3702Δ*	0.86	0.14	0.74	0.26
*swc2Δ*	0.74	0.26	0.50	0.50
*swr1Δ*	0.75	0.25	0.56	0.44
*tea1Δ*	0.62	0.38	0.52	0.48
*tea2Δ*	0.65	0.35	0.39	0.61
*tea4Δ*	0.61	0.39	0.34	0.66
*tip1Δ*	0.68	0.32	0.43	0.57
*vps71Δ*	0.63	0.37	0.58	0.42
wild-type	0.94	0.06	0.93	0.07

1 represents the percentage of straight cells that, in each mutant, remained straight; 2 denotes the percentage of straight cells that changed from straight to curved (StoC); 3 represents the percentage of curved cells that evolved to straight (CtoS); 4 shows the percentage of curved cells that remained curved.

### Inheritance rules predict the existence of multiple pathways controlling axial cell growth

Our results with curved mutants suggest that there are at least 16 genes involved in persistent axial cell growth regulation in this species, which when knocked out give rise to different modes of shape inheritance. However, we found that knockouts of similar machineries seem to have similar inheritance frequencies (Table 2). Therefore, we reasoned that grouping mutants by a combination of the various inheritance rules we quantitated might allow grouping cell shape regulatory machineries in pathways and might clarify the role of genes never before associated with mophogenetic control.

**Figure 3:**
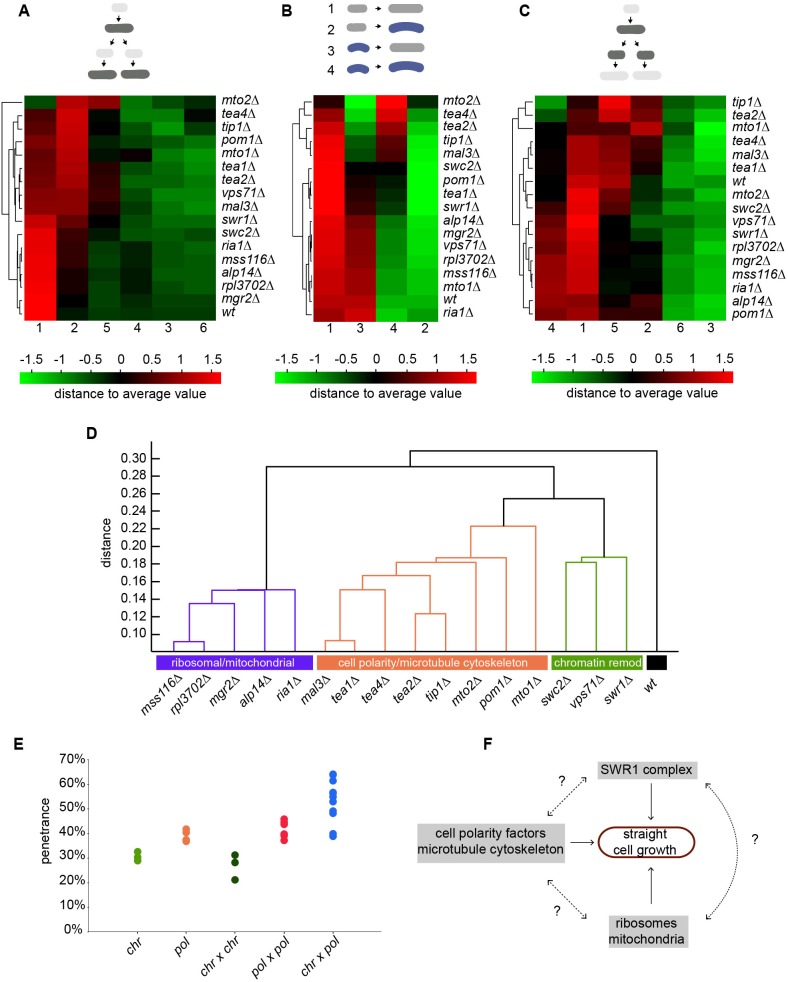
Cell shape inheritance rules predict the existence of multiple pathways controlling axial cell growth. **A–C**. Clustergrams of all ‘curved’ mutants based on different inheritance frequencies quantitated: cell shape inheritance comparing the phenotype of mothers before septation with that of daughters before septation (**A**; using the six frequencies described in Fig. 1D); shape changes during growth (**B**; 1–4 represent, respectively, the frequencies of straight cells that remain straight after growth, straight cells that become curved, curved cells that straighten and curved cells that continue being curved); and cell shape inheritance comparing the phenotype of mothers before septation with that of daughters directly after septation, when they are born (**C**; the frequencies 1–6 follow the same logics as in A). The clustergrams display in a colour scale (black: average, red/green: higher/lower than the average) the different frequencies for each and order them hierarchically in a dendrogram based on their level of similarity. **D**. *Superdendrogram* grouping the sixteen curved mutants based on their similarities in cell shape inheritance pattern, combining all features quantitated (including cell shape and growth pattern, inheritance pattern over two generations, cytokinesis defects and cell shape changes during growth, see text for details). Three distinctive groups - ‘mitochondrial/ribosomal’, ‘cell polarity/microtubule cytoskeleton’ (‘pol’) and ‘chromatin remodelling’ (‘chr’) - and the wild-type are obtained using a cut-off at a distance of 0.24 (arbitrary units). **E**. Comparison of the overall penetrances of single (‘chr’: *swc2Δ*, *swr1Δ* and *vps71Δ*; ‘pol’: *tea1Δ*, *tea2Δ*, *tip1Δ* and *tea4Δ*) and double curved mutants (‘chr×chr’, ‘pol×pol’ and ‘chr×pol’; generated by the combination of each of the aforementioned single mutants). Penetrances of single and double mutants of the same group were not significantly different (p = 0.348 for the ‘chr’ group; p = 0.209 for the ‘pol’ group). By contrast, penetrances of double mutants from different groups were significantly different from the former (p = 0.023 for ‘chr×chr’ against ‘pol×pol’; p = 0.001 for the other two). **F**. Schematic representation of the different machineries that control axial cell growth in *S. pombe*.

Thus, we clustered independently each of the different datasets obtained in Tables 2–4 and Fig. 2B (Fig. 3A–C; where every time only one of the various features of cell shape inheritance was used for clustering), computed for each clustergram the normalized distances between gene pairs, and then we integrated all those distances into a *superdendrogram* combining all experiments together (Fig. 3D). Interestingly, this strategy naturally grouped mutants into three distinct clusters, each enriched in functionally-linked subsets of genes. One cluster consisted of *swc2Δ*, *swr1Δ* and *vps71Δ*, and corresponds to genes encoding proteins that belong to a chromatin remodelling complex (termed ‘chromatin group’ from now on). A second cluster comprised *tea1Δ*, *tea2Δ*, *tea4Δ*, *mal3Δ*, *tip1Δ*, *mto1Δ*, *mto2Δ* and *pom1Δ*, corresponding to genes involved in cell polarity and cytoskeletal organization (‘polarity group’). A third heterogeneous yet distinct cluster included mutants in genes ontologically apparently unrelated to each other: *mgr2* and *mss116* (mitochondria), *alp14* (microtubule nucleation) and *ria1* and *rpl3702* (ribosomal biogenesis). The wild-type constituted a fourth singleton cluster (Fig. 3D). Interestingly, although as expected mutants from the polarity group had obvious aberrant microtubules and polarity landmark distribution (as assessed by expression of Atb2-mCherry and Tea1-3GFP, Figure S5; notice the example of an *mto2Δ* cell, in which these aberrations seem to cause cell curvature) mutants from the other groups did not display any obvious such defects (Figure S5), suggesting that the means by which curved cells form in those mutants is different from the currently proposed models of *S. pombe* cell bending [16]. As actin is itself part of the SWR1 complex [17], we investigated in addition whether the localization of the actin cytoskeleton was affected in the chromatin group. We found that neither actin cables nor actin patches displayed observable defects in SWR1 mutants (Figure S6). This suggests that the way this complex controls cell shape maintenance is likely not via directly controlling actin.

Finally, we tested whether the contribution of those machineries to axial growth is additive, by crossing the three mutants of the chromatin group against four mutants of the polarity group and measuring the penetrance of all combinations of double mutants, thus testing for epistatic relationships between the genes (see Fig. 3E and Table S4 for details). We found that although mutation of two genes belonging to only one group displayed a similar penetrance as single mutants in that group, deletion of two genes from the two different groups produced a significantly stronger deregulation of cell shape (Fig. 3E; p = 0.001 in both cases), displaying a higher number of curved cells and in some cases even of ‘T-shaped’ cells (*swr1Δ tea4Δ*, *swc2Δ tea4Δ* and *swc2Δ tea1Δ*).

Taken together, these results indicate that there are at least two complementary, separable pathways that control persistent axial cell growth, a known one corresponding to cytoskeletal and cell polarity regulators and one involving chromatin remodelling factors. Although impairment of each apparently causes an apparently similar deregulation, the way that deregulation is transmitted to the progeny keeping a characteristic population homeostasis is different. This suggests the existence of multiple, separable machineries and pathways regulating that specific feature of morphogenetic control in this species (Fig. 3F).

## Discussion

The aim of this work was to clarify the impact that the general morphological properties of a cell have on the shape and growth pattern of its progeny, by investigating morphogenetic inheritance in the spherocylindrically-shaped and unicellular *S. pombe* [18]. Although extensive work in *S. pombe* has addressed the regulation of polarity establishment and maintenance through the cell cycle [16], [19]–[24] and some work has investigated how mechanically-imposed changes in cell geometry induce cytoskeletal re-organization [25], [26], how *S. pombe* is able to dynamically maintain shape throughout generations is not fully understood. In particular, it is unclear whether its characteristic rod shape is predominantly consequential or whether instead it plays an active role in defining cell shape fate. We find that *S. pombe* cell shape is actively modulated by multiple factors that impact on growth pattern throughout generations, in a way that varies depending on the morphogenetic cascades involved. Cell shape transmission seems to follow, to a first approximation, first order Markovian dynamics leading to a stable homeostatic penetrance of shape phenotype. We chose to focus on curved shape mutants as they constitute a class of mutants that have been traditionally understudied yet that produce a high frequency of misshapen cells (Fig. 1 and Table 1). A deviation in the 180° growth axis is, albeit subtle, a relatively common defect in *S. pombe*, while more conspicuous mutant phenotypes such as T-shape occur at a much lower frequency (<1% in exponential standard culture conditions; [41]–[43]). Although we have focused here on that particular phenotypic mutant class, we think that the general qualitative traits of phenotypic transmission observed here are likely applicable to phenotypic transmission as a whole in *S. pombe*, and potentially in other cell types.

What gives rise to individual straight/curved cells giving rise to one particular cell division outcome versus another? One possibility is the non-exact equipartition of cellular material between daughter cells at cell division. Fission yeast growth pattern is not totally symmetrical [6] and as a result all cell ends might not have the same growth history and composition, leading to uneven repartition at cytokinesis of polarity landmarks, secretory machinery, cell wall composition/properties or even some organelles. Similarly, chromatin structure and gene expression/silencing patterns might differ between cells, in a way that affects morphogenetic control differently on a cell-by-cell basis. In this manner each cell might possess at birth a unique combination of intracellular and geometrical inherited properties that might be reinforced, perpetuated or diluted through active mechanisms and/or by noise. This in turn might allow the cell to overcome or not the morphogenetic thresholds in shape fate set by its ancestors. We have evidence from our results that such mechanisms exist. For example, curved-born cells very often straighten their shape during growth (‘dilution’) and straight-born cells curve gradually as they may accumulate certain defects during their cell cycle (‘reinforcement’). By contrast, we have observed that most impaired cells do not overcome their defects during only one cell cycle. This might be due to the existence of limiting factors, such as a limited turnover of cell wall and of proteins involved in setting the growth directionality, or due to the dominance of cell size regulation which strictly triggers cell division when the cells reach ∼14 µm in length [23]. Thus, the dynamical nature of cell shape inheritance may reflect that there are two complementary layers governing the establishment and regulation of stable axial growth in *S. pombe*: 1) static contributions provided by inheritable factors, for example the cell wall, which might locally keep a transient shape template and 2) dynamic contributions which give the growth process ‘plasticity’. Such a strategy could be optimal to maintain cellular architecture and shape at the level of the entire cell population (where heterogeneity could be favourable for buffering/responding to environmental changes) as well as intracellularly (where it may be disadvantageous in terms of cell economy to re-establish cell architecture each time from scratch). This is akin at the global cellular level to the balance between ‘self-assembly’ and ‘self-organization’ mechanisms observed in the formation of subcellular structures such as the mitotic spindle, where the combination of both types of mechanisms impart to cells a balance between reliable structure formation and plasticity [27].

We have found that the cell wall indeed keeps a transient local template of cell shape that is inherited differently for different mutants and genotypes. This role of the cell wall is similar to what has been described for *Saccharomyces cerevisiae*, where the cellular budding pattern depends, among other factors, on the location of pre-existent budding scars in the cell wall [28] and to what has been proposed for cell adhesion patterns and the ECM in animal cells. In those cells, the ECM is thought to be a determinant for cellular fate during tissue/organ differentiation and repair [29]–[31] likely by influencing intracellular machineries [32], [33].

Our analysis suggests that there are multiple, separable groups of genes participate in regulating fission yeast axial cell growth (Fig. 3D–F). A first group includes the vast majority of factors classically associated with polarity and cytoskeletal regulation and which (as shown in Figure S5) when deleted can give rise to cytoskeletal or polarity defects: the kelch repeat protein Tea1 and its interactor Tea4, which are deposited by microtubules at the cell cortex where they serve as landmarks for polarized secretion via recruitment of the formin For3, an actin cable nucleator [19], [34]; the +TIP Tip1, which localizes at the microtubule plus ends binding Tea1/Tea4 and facilitating their delivery to the cortex; the kinesin Tea2, which transports Tea1/Tip1 to microtubule plus ends and which, in turn, is loaded onto the microtubules by the EB1 homologue Mal3 [35]; the centrosomin-related factor Mto1 and its interactor Mto2, which participate in cytoplasmic nucleation of microtubules [10], [36]; and the cell size control-related dual-specificity tyrosine phosphorylation-regulated kinase (DYRK) Pom1 [37], which is kept at the cell ends in a Tea4-dependent manner [38]. Although deletion of the genes encoding those proteins is known to generate curved/bent cells [7], [10], [14], [35], [39]–[41], we find that they also affect in a very similar manner cell shape maintenance within and across generations in a way that is distinct from that of other curved mutants.

A second group includes the *S. pombe* homologues of the chromatin remodeling factors Swc2, Swr1 and Vps71 (also called Swc6) [17]. All of those factors belong to the conserved multiprotein SWR1/SRCAP complex, whose role is to replace in nucleosomes located at heterochromatic regions the canonical histone H2A with variant H2A.Z, enhancing transcription of otherwise repressed genes [17], [42]. A relationship between those mechanisms and cell morphogenesis has only been vaguely reported. In budding yeast, where this complex has been more extensively studied, mutations in *arp4* (which encodes one complex subunit) cause changes in cell shape and size and it has been hypothesized that those defects occur via upregulation of stress genes responsible for cell wall integrity [43], [44], possibly due to the incorporation of H2A.Z in incorrect locations. In mammals, skeletal muscle differentiation fails and cells accumulate morphological defects when the homologue of *vps71*, p18^Hamlet^, is suppressed [45], due to the impairment of the induction of several late muscle differentiation-specific genes as myosin heavy chain (MHC) and muscle creatine kinase (MCK). In *S. pombe*, mutations in the SWR1 complex have been shown to produce misincorporation of H2A.Z to subtelomeric regions, which comprise the lowest expressed genes during vegetative growth and are especially rich in meiotic-specific genes [46], and though it has been shown that *swr1Δ* and *swc2Δ* enhance the cell shape defects of γ-tubulin complex mutants [47] (a result in agreement with our epistasis results), gene expression analysis of those mutants did not find any morphogenesis- or cytoskeleton-related genes whose expression was considerably reduced or enhanced [47]. In addition to this, we observed that these mutants do not display any apparent aberration in the microtubule cytoskeleton, in the distribution of the polarity factor Tea1 (Fig. S5) or in the localization of actin patches and actin cables (Fig. S6). Thus, the exact mechanistic relationship of this complex with morphogenetic control will have to be clarified.

A third group of genes consists of the 60S ribosomal protein Rpl3702 (also called L37) [48] and the ribosome-related GTPase Ria1 [49], the mitochondrial RNA helicase Mss116 [50] and the mitochondrial membrane protein Mgr2 [51] (all of them are uncharacterized in fission yeast with their role only inferred *in silico;* www.pombase.org), and the TOG-related and mitotic spindle pole body-associated microtubule regulator Alp14 [52]. Although we cannot rule out the possibility that this heterogeneous group of factors was clustered because of the low penetrance of their mutants or the presence in some of the mutants of features undetectable by our analysis (for example, *alp14Δ* shows a relatively high percentage of bent cells and often its cells fail to divide), we could hypothesize that the segregation of organelles and other intracellular components (such as ribosomes or mitochondria) at cell division might be subjected to a common regulation, giving rise to similar cell shape transmission rules. For example, the association of mitochondria with spindle pole bodies during spindle elongation has been proposed to be important for correct organelle inheritance in this species [53], [54]. Future work will need to investigate why impairment of those ribosomal and mitochondrial components affects cell shape and what links their mode of action.

Why persistent axial cell growth should rely on multiple layers of control is an interesting question. One possibility is that this allows cells to adapt and accommodate to a diversity of environmental conditions which might alter axial growth pattern, like physical barriers (imposed by the environment or other cells), and that the different machineries impart robustness to axial growth. Alternatively it might allow cells to deviate from that pattern of growth selectively under certain conditions, such as mating or depletion of certain nutrients [55]. Future work should focus on testing these possibilities and clarifying how the different machineries that secure persistent axial growth interact at a molecular level.

Interestingly, a consequence of the finding that multiple pathways control a common phenotypic outcome is that it implies that static cellular shape signatures alone are likely not sufficient for inferring morphological networks from microscopy-based phenotypic screens [56]. Conversely, our findings indicate that dynamical morphogenetic features can be used to infer morphogenetic network information even from single gene knockout data [57]. This may make such features particularly attractive in the context of genome-wide morphogenesis screens using yeast or other cell types like *D. melanogaster* S2 cells, as they might provide a means for obtaining some of the network information which could otherwise only be obtained with synthetic genetic interaction analysis by double RNAi [58].

## Materials and Methods

### Strains, media and image acquisition and analysis

All the strains used in this study are listed in Table S1. The single deletion mutants come from the commercially available “*S. pombe* Haploid Deletion Mutant Set version 2.0” strains collection (Bioneer Corporation; http://pombe.bioneer.com) [59], except *mto2Δ*. The cassette used to substitute *mto2* by a hygromycin B (*hph*) resistance selection marker was generated by PCR, following the procedure described in [60]. To generate double mutants we used standard crossing methods [61], although for the ones involving *tea1Δ*, *tea2Δ*, *tea4Δ*, *vps71* and *tip1Δ* we used the PEM-2 system, developed by [62]. All the strains were checked by PCR.

Cells were cultured and kept in exponential growth for 48 hours at 32°C in rich YES medium (‘Yeast Extract with Supplements’) before being treated and/or imaged. For imaging we used 35 mm Glass Bottom plates (MatTek corporation), pre-coated with 5 µl lectin (1 mg/ml; Sigma; L1395 and Patricell Ltd; L-1301-25). Images were acquired with a DeltaVision system (Applied Precision/GE Healthcare) comprising an Olympus IX81 epi-fluorescence inverted microscope, Olympus UPlanSapo ×100 and ×60 oil immersion lenses (numerical aperture 1.4 and 1.42, respectively) and 1.512 refractive index immersion oil (Applied Precision), and using the proprietary software SoftWoRx. Quantum dots (Qdots; Qdot 605 Streptavidin Conjugate) were imaged with a 580 nm dsRed standard filter set, whereas cells expressing Tea1-3GFP and Atb2-mCherry were imaged with the FITC and TRITC filter sets using the DeltaVision specific tool “optical axis integration” (OAI). Image processing was done using SoftWoRx and the open-source software Fiji (http://fiji.sc/wiki/index.php/Fiji).

### Study of cell curvature inheritance and penetrance measurement

To follow cell lines during several generations, we layered 30 µl of cell culture (OD = 0.2) onto a lectin-covered MatTek plate coverslip and let cells attach to the coverslip for 10 minutes at room temperature. Afterwards, we washed out non-attached cells with YES containing 0.5% low melting point (LMP) agar kept at 32°C, subsequently covered the plate and cells with 3 ml YES with 1.5% LMP agar (32°C), and waited until the YES LMP gelled. This constrained cells to grow and divide as a monolayer 2-dimensionally and in the plane of focus. For each of the genotypes mentioned in the text, 20–40 parental cells and their progeny were then imaged for approximately four generations, every 10 minutes for 10 hours at 30°C, using transmitted light microscopy (under these conditions wild-type cells double every ∼2.2 hours), by z-stack (focal plane separation: 0.5 µm) time-lapse acquisition.

Cell wall tracking for multiple generations in live cells was conducted using streptavidin conjugated 605 nm emission fluorescent quantum dots (Qdots) (Invitrogen). First, cells were incubated for 10 minutes in a dilution 1∶5000 of biotinylated isolectin in YES at room temperature (isolectin GS-IB4 from *Griffonia simplicifolia*, biotin-XX conjugate; Invitrogen; biotin and streptavidin form one of the strongest non-covalent bonds in nature [63], whereas lectins have been extensively documented to bind yeast cell wall mannan [64]). After three washes with YES medium, cells were incubated 10 minutes in a dilution 1∶500 Qdots:YES medium at the same temperature. Finally, cells were imaged as described in the previous paragraph. In this case, images were acquired every 30 minutes for 9 hours at 30°C in both transmitted light and the TRITC fluorescence channel.

Calculation of cell curvature for each mutant was done using a custom-written Fiji macro, to analyze images and registered image sequences. The macro keeps track of each cell of a lineage and estimates the curvature of each cell by calculating the inverted radius of curvature of a circle crossing both cell end apices and the cell middle (radius^−1^; see Figure 1B for details). With that method, a perfectly straight cell was assigned radius^−1^ = 0, and cells were considered to be curved above radius^−1^ = 0.028 µm^−1^ based on a threshold set by visual inspection. Through growth, fluctuations in cells’ curvature could be observed (Fig. S3). Because it was not obvious to us how to analyze those fluctuations and whether any useful information could be contained in them, we instead took a simpler approach and chose to measure cells’ curvature only at two time points: just after cytokinesis and before the septum became obvious in the light microscopy channel. The data generated was exported and then analyzed using custom-written MatLab (MathWorks) functions to compute changes in shape and inheritance events, and calculate the frequencies of each event (Fig. 1D). Correct computational assignment of a visually curved cell as curved occurred in more than 90% of cases for short cells and long cells. Because our curvature quantitation strategy was not appropriate to analyze S-shaped cells (typically less than 3% of cells for most mutants; see Table S2), we intentionally eliminated lineages containing “S” shapes from our analysis.

For the measurements of curvature along the cell perimeter shown in Fig. S4, we used a modification of a MatLab routine (kind gift of Jacques Dumais, Universidad Adolfo Ibáñez, Viña del Mar, Chile [65]), which, based on the transmission light channel images, detects the 1 pixel-wide outline of the cell, finds fiducial markers on it and calculates, for the whole cell outline, the radius of curvature of each triplet of markers in microns^−1^.

The measured ‘overall penetrance’ was the percentage of all curved cells present in a population. The measured ‘penetrance at septation’ was the fraction of curved cells amongst all cells about to septate, as assessed visually from time-lapse movies. This distinction is made explicit in the figure legends but not in the main text, for simplicity.

Data referring to cellular dimensions and cell cycle duration (time between cell birth and division) were calculated manually. To find the penetrance (percentage of curved cells in the population) of each mutant, we measured from 200 to 500 cells with the Fiji macro. Only penetrance ( =  percentage of curved cells), and not expressivity ( =  the radius of curvature of individual cells), was used for our analysis.

Regarding the cell shape inheritance rules, they were set in a ‘mother-daughter’ (2 generations) or in a ‘grandmother-granddaughters’ (3 generations) basis but, in order to obtain a statistically considerable amount of data, we measured individual lineages over 4 generations. On one hand this could give us, per movie, the information for 7 divisions and 15 cell growth periods; on the other hand, it allowed us to deal with the fact that we could not always track the whole cell cycle of the first cell of the lineage.

### Markovian modelling of growth inheritance and predicted penetrance

To model shape inheritance, we assumed that the number of curved (c) and straight (s) cells at generation n, 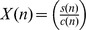, depends only on 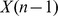
and the transition matrix 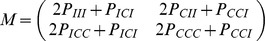, with 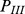 the probability for a straight cell (I) to give two straight cells (II) - which would correspond to the frequency ′1/(1+2+3)′ of Table 2 -, 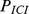 the probability for a straight cell (I) to give a curved and a straight cell (CI) - which would correspond to the frequency to ′2/(1+2+3)′ of Table 2 -, etc. We could then write 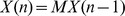. As is usual in the study of dynamical systems, 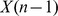 to study long-term behaviour of the system, one can look into the equivalent continuous system of differential equations of the form 
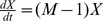
. The analytical solution to that system is known and is the sum of two exponential growth terms, as expected. The limit for 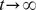 gave us a simple expression for the predicted penetrance at equilibrium: 
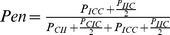
. This also ensured that the model led to a stable, fixed homeostatic equilibrium.

### Cluster analysis

Clustering analysis was done using hierarchical clustering with Euclidean distance metric and average linkage. It produced dendogram trees which, when thresholded, gave rise to the set cluster. The first three panels A, B and C of Figure 3 correspond to three different sets of transition frequencies (see figure legend for details). To generate the *superdendrogram* of Fig. 3D we calculated a global distance between each mutant in a given dataset based on all their frequencies of shape inheritance or changes during growth. The datasets used for calculating that distance were the frequencies of ‘changes during growth’ (Table 4), the frequencies of cell shape inheritance considering cells before septation (Table 2) and considering cells after division, the frequencies of direct cell shape inheritance (Table 3) and the penetrance of the progenies of curved and straight ancestors after two generations (Fig. 2G). The distances obtained were normalized in each dataset (so the maximum distance between two mutants became 1) before calculating the mean of the distance between each mutant. Those average distances were then treated as inputs to generate a *superdendrogram*, as above. All the computations were done in MATLAB, using functions provided in the bioinformatics and statistics toolboxes.

To check the sensitivity of the *superdendrogram* analysis results to the datasets used, we redid the analysis using two other methods. The first method used only the distances obtained from the datasets represented in Figs. 3B and 3C (‘growth’ and ‘cytokinesis’, respectively) to generate the *superdendrogram*. The second method separated the five different datasets into two main groups - one comprising the datasets from Figs. 3A, 3B and 2G (group 1), and another comprising the other two datasets (group 2) - by looking at pairwise correlations across the datasets and grouping highly correlated datasets together. Groups 1 and 2 were then used to generate a *superdendrogram*. In both cases, the same functional groups of genes displayed in Fig. 3D were identified (not shown). This confirms the robustness of the clustering analysis.

## Supporting Information

Figure S1 Transmission light images of the 16 curved mutants addressed in our study and the wild-type. The yellow asterisk in the *tea1Δ* image highlights a cell that shows simultaneously bent and curved morphology. Bars, 10 µm.
(PDF)

Figure S2 Representation of the degree of curvature (average curvature of the curved cells in the population) of each mutant against its penetrance (percentage of curved cells in the population). The horizontal lines show the standard deviation of the expressivity. The number of cells measured varied depending on the mutant, from 159 to 420.
(PDF)

Figure S3 Evolution of the curvature of the cells of a *tip1Δ* (top; images of this lineage are displayed in Fig. 1C and in Movie S1) and a wild-type (bottom; this lineage corresponds to Movie S6) lineages. The measurements were carried out each 10 minutes and the first time frame after cytokinesis was discarded. The horizontal dotted red line marks the threshold used to discriminate curved from straight cells (0.028 µm^−1^).
(PDF)

Figure S4 Examples of changes in cell morphology during growth. A *tea1Δ* monopolar straight cell and a *tip1Δ* monopolar curved cell were imaged during their whole cycle of growth. Images showing their morphology at the beginning of the movie and after 30, 60 and 90 minutes are displayed on the top. The panels in the middle represent the evolution of the cell outline curvature during growth. For both cells the peak on the left corresponds to the non growing end, whereas the peak on the right represents the evolving growing end. A negative curvature indicates a concavity in the cell outline. Superpositions of the successive time frames are displayed at the bottom to show the contrast between the maintenance of inherited structures and the constant morphological evolution of the growing tips. Bars, 5 µm.
(PDF)

Figure S5 Distribution of the microtubule cytoskeleton and a polarity factor in curved mutants. **A)** Images of cells of 12 of the curved mutants and the wild-type that express Atb2-mCherry and Tea1-3GFP. The images were acquired via optical axis integration (OAI), which summed in a single frame all the information contained inside the cell (separation between top and bottom of the sample: 5 µm). The arrows in the *tea4Δ* image point abnormally high concentrations of Tea1-3GFP at the non growing end. **B)** Image sequence of an *mto2Δ* cell curving after misplacing Tea1-3GFP at the tips through its aberrant unique Atb2-mCherry microtubule bundle. Bars, 5 µm.
(PDF)

Figure S6 Distribution of the actin cables and patches in curved mutants. Images of cells of 12 of the curved mutants and the wild-type that express GFP-lifeact. The images are maximal intensity projections of Z-stacks (separation between the 27 Z-planes: 0.2 µm).
(PDF)

Table S1 Strains used in this study.
(DOCX)

Table S2 Compilation of all the measurements of the studied curved mutants and the wild-type. This file allows the reader to sort and filter the data according to every set of data.
(XLS)

Table S3 Detailed information of a specific tip1Δ lineage across four generations. The column on the left shows the cells’ names. Each cell, except the first one (*1) contains two sets of data, represented in two rows. The first one (from the top to the bottom of the table) corresponds to the measurements taken after the mother’s cell division, whereas the second one shows the measurements taken before the cell started septating. These data belong to the lineage shown in Fig. 1C and Movie S1.
(DOCX)

Table S4 Overall penetrance of the curved phenotype in each of the strains listed. The number of cells measured varied depending on the mutant, from 112 to 497. Thus, cell shape is modulated by multiple and complex factors - cell wall inheritance, cell shape changes at division, active growth pattern changes, and likely many others -, which together give rise to the overall cell shape inheritance rules specific to each genotype.
(DOCX)

Movie S1 Time-lapse movie showing the lineage of a single *tip1Δ* cell over several generations. 43 Z-stacks covering a sample thickness of 6 µm (with a focal plane separation of 0.5 µm) were acquired every 10 minutes in the transmitted light channel during 420 minutes. The images shown are maximal intensity projections of the 2 equatorial microns of the Z-stacks after image registration. Bar, 10 µm.
(MOV)

Movie S2 Time-lapse movie showing the lineage of a single *tea4Δ* cell over several generations. 59 Z-stacks covering a sample thickness of 6 µm (with a focal plane separation of 0.5 µm) were acquired every 10 minutes in the transmitted light channel during 580 minutes. The images shown are maximal intensity projections of the 2 equatorial microns of the Z-stacks after image registration. Bar, 10 µm.
(MOV)

Movie S3 Time-lapse movie showing the lineage of a single *vps71Δ* cell over several generations. 57 z-stacks covering a sample thickness of 6 µm (with a focal plane separation of 0.5 µm) were acquired every 10 minutes in the transmitted light channel during 560 minutes. The images shown are maximal intensity projections of the 2 equatorial microns of the Z-stacks after image registration. Bar, 10 µm.
(MOV)

Movie S4 Time-lapse movie showing the lineage of a single *pom1Δ* cell over several generations. 42 Z-stacks covering a sample thickness of 6 µm (with a focal plane separation of 0.5 µm) were acquired every 10 minutes in the transmitted light channel during 410 minutes. The images shown are maximal intensity projections of the 2 equatorial microns of the Z-stacks after image registration. Bar, 10 µm.
(MOV)

Movie S5 Time-lapse movie showing the lineage of a single *mss116Δ* cell over several generations. 53 Z-stacks covering a sample thickness of 6 µm (with a focal plane separation of 0.5 µm) were acquired every 10 minutes in the transmitted light channel during 520 minutes. The images shown are maximal intensity projections of the 2 equatorial microns of the Z-stacks after image registration. Bar, 10 µm.
(MOV)

Movie S6 Time-lapse movie showing the lineage of a single wild-type cell over several generations. 56 Z-stacks covering a sample thickness of 6 µm (with a focal plane separation of 0.5 µm) were acquired every 10 minutes in the transmitted light channel during 550 minutes. The images shown are maximal intensity projections of the 2 equatorial microns of the Z-stacks after image registration. Bar, 10 µm.
(MOV)
