# Supplementary figures and images for: Dynamics of Cell Shape Inheritance in Fission Yeast
Source: PLoS One. 2014 Sep 11;9(9):e106959. doi: 10.1371/journal.pone.0106959 (PMC4161360; doi:10.1371/journal.pone.0106959)

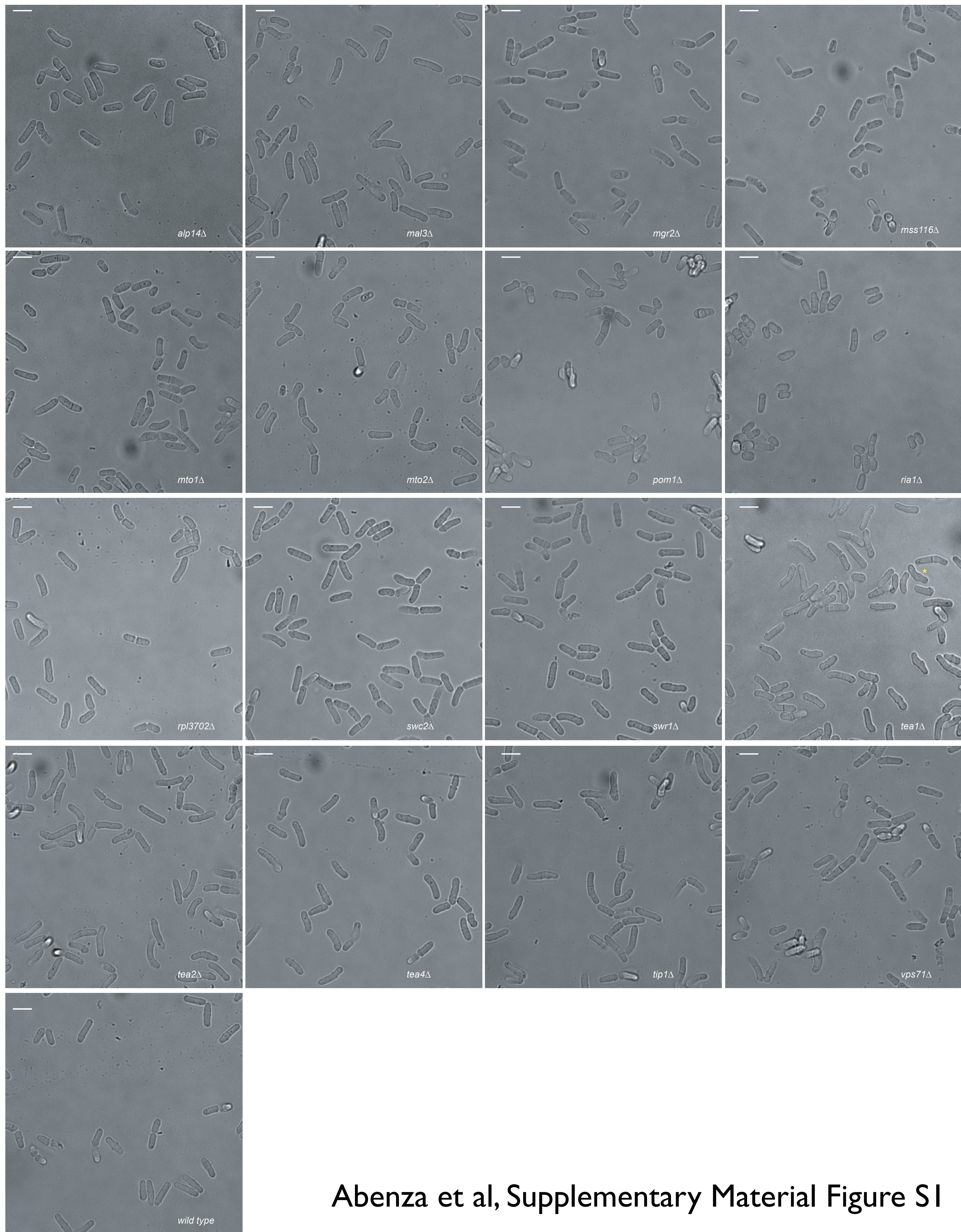

Abenza et al, Supplementary Material Figure S I

Supplement: Figure S1 — Transmission light images of the 16 curved mutants addressed in our study and the wild-type. The yellow asterisk in the tea1Δ image highlights a cell that shows simultaneously bent and curved morphology. Bars, 10 µm. (PDF) [file pone.0106959.s001.pdf]

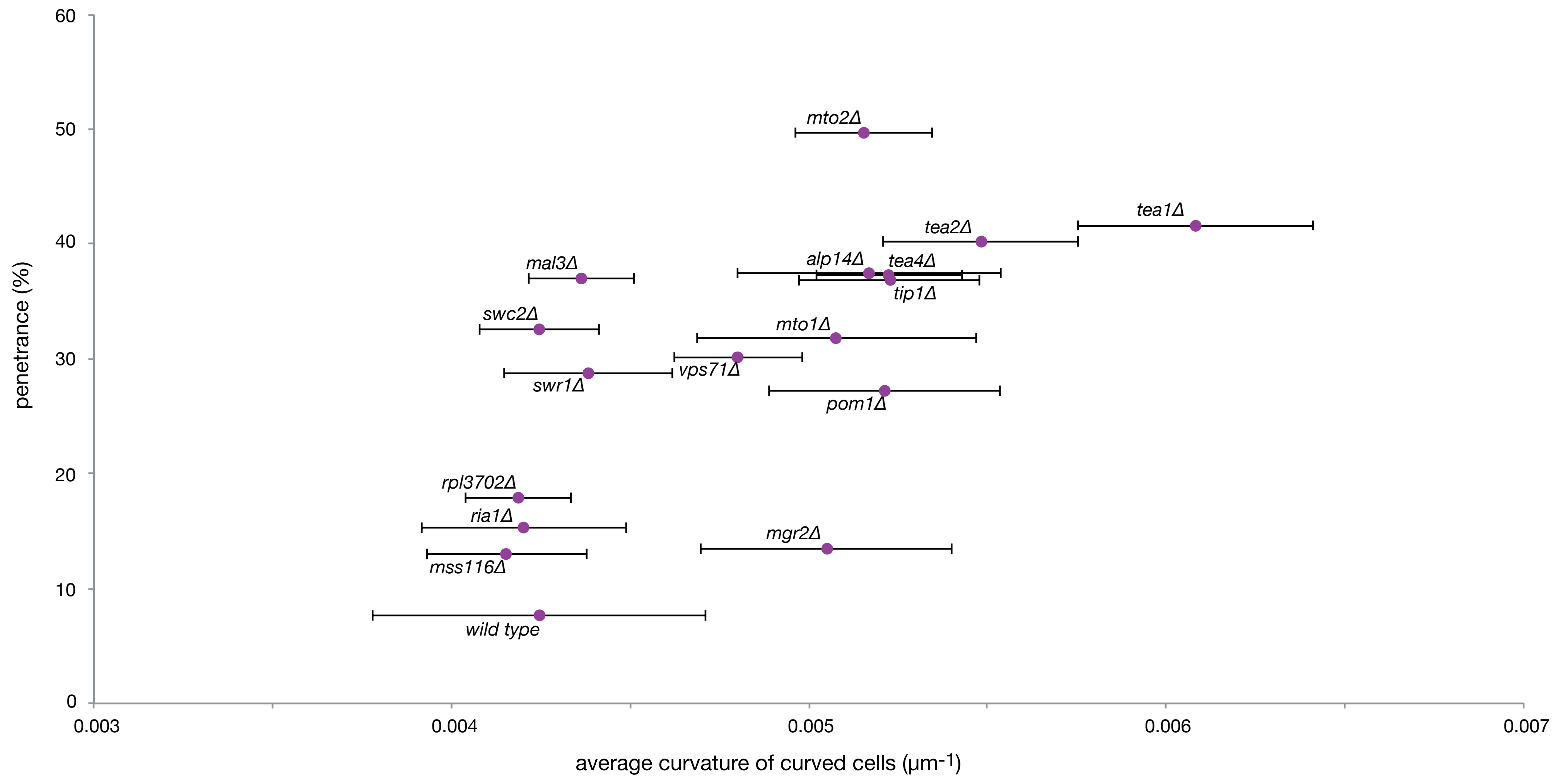

Supplement: Figure S2 — Representation of the degree of curvature (average curvature of the curved cells in the population) of each mutant against its penetrance (percentage of curved cells in the population). The horizontal lines show the standard deviation of the expressivity. The number of cells measured varied depending on the mutant, from 159 to 420. (PDF) [file pone.0106959.s002.pdf]

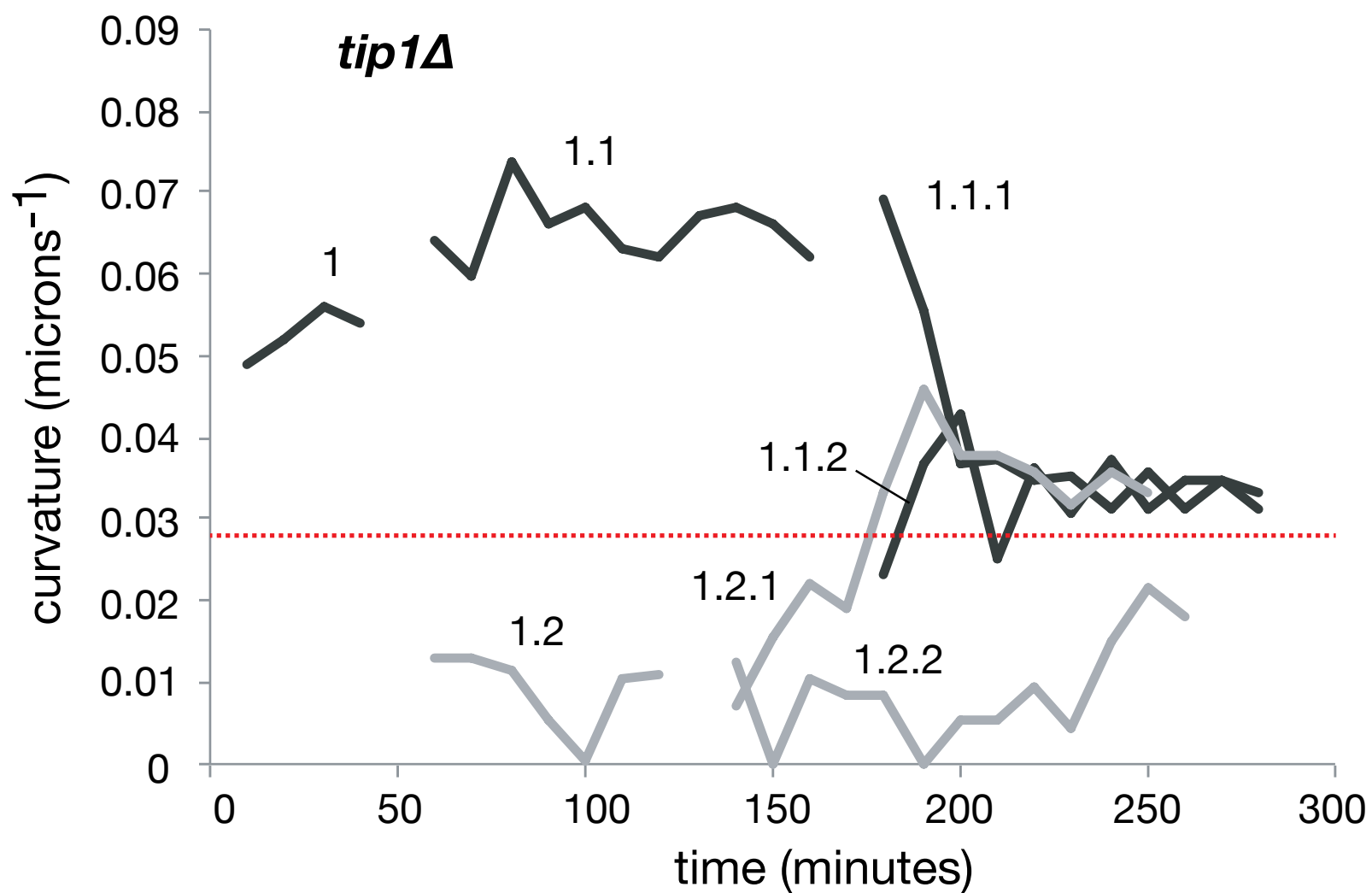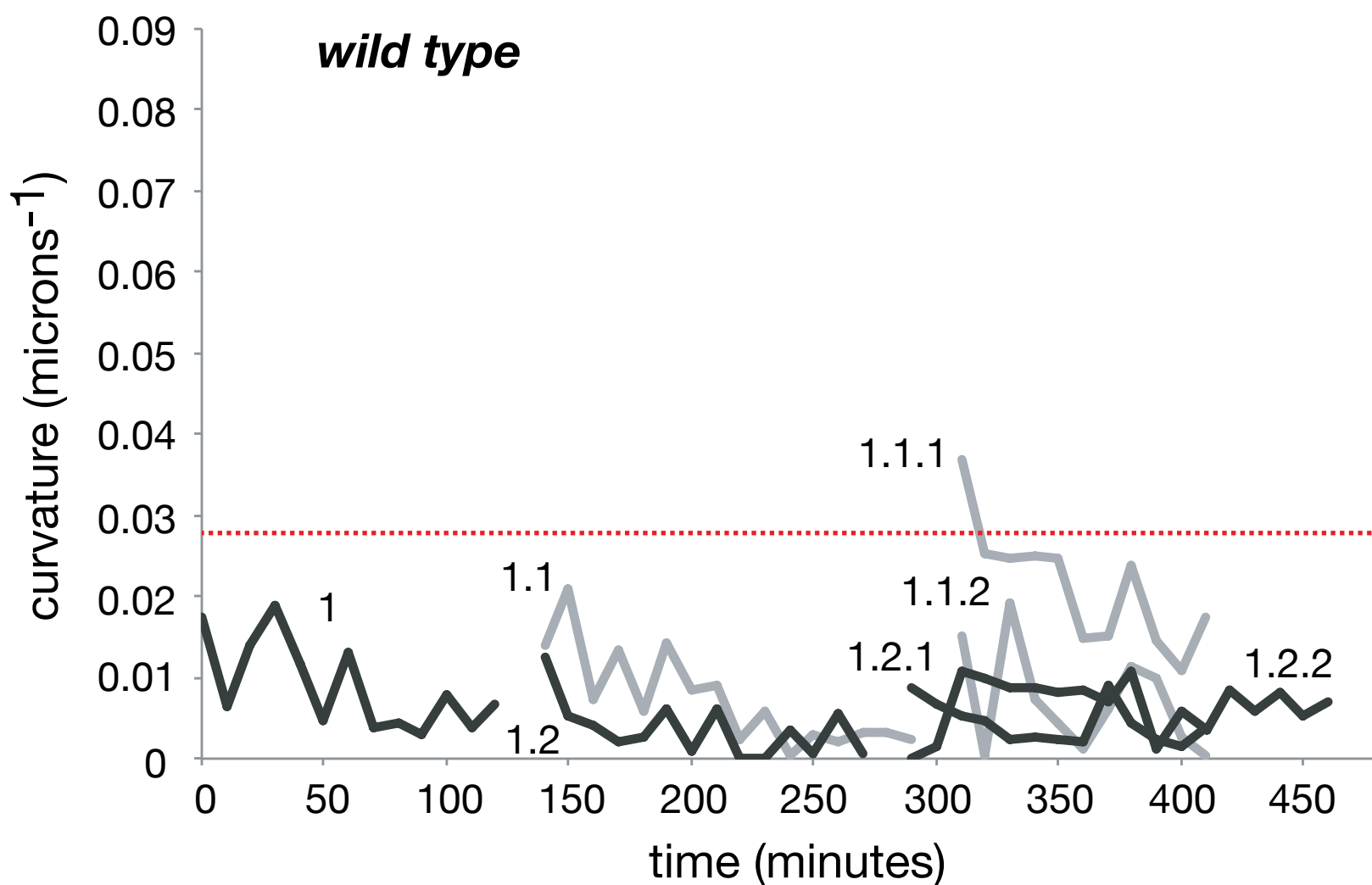

Supplement: Figure S3 — Evolution of the curvature of the cells of a tip1Δ (top; images of this lineage are displayed in Fig. 1C and in Movie S1) and a wild-type (bottom; this lineage corresponds to Movie S6) lineages. The measurements were carried out each 10 minutes and the first time frame after cytokinesis was discarded. The horizontal dotted red line marks the threshold used to discriminate curved from straight cells (0.028 µm−1). (PDF) [file pone.0106959.s003.pdf]

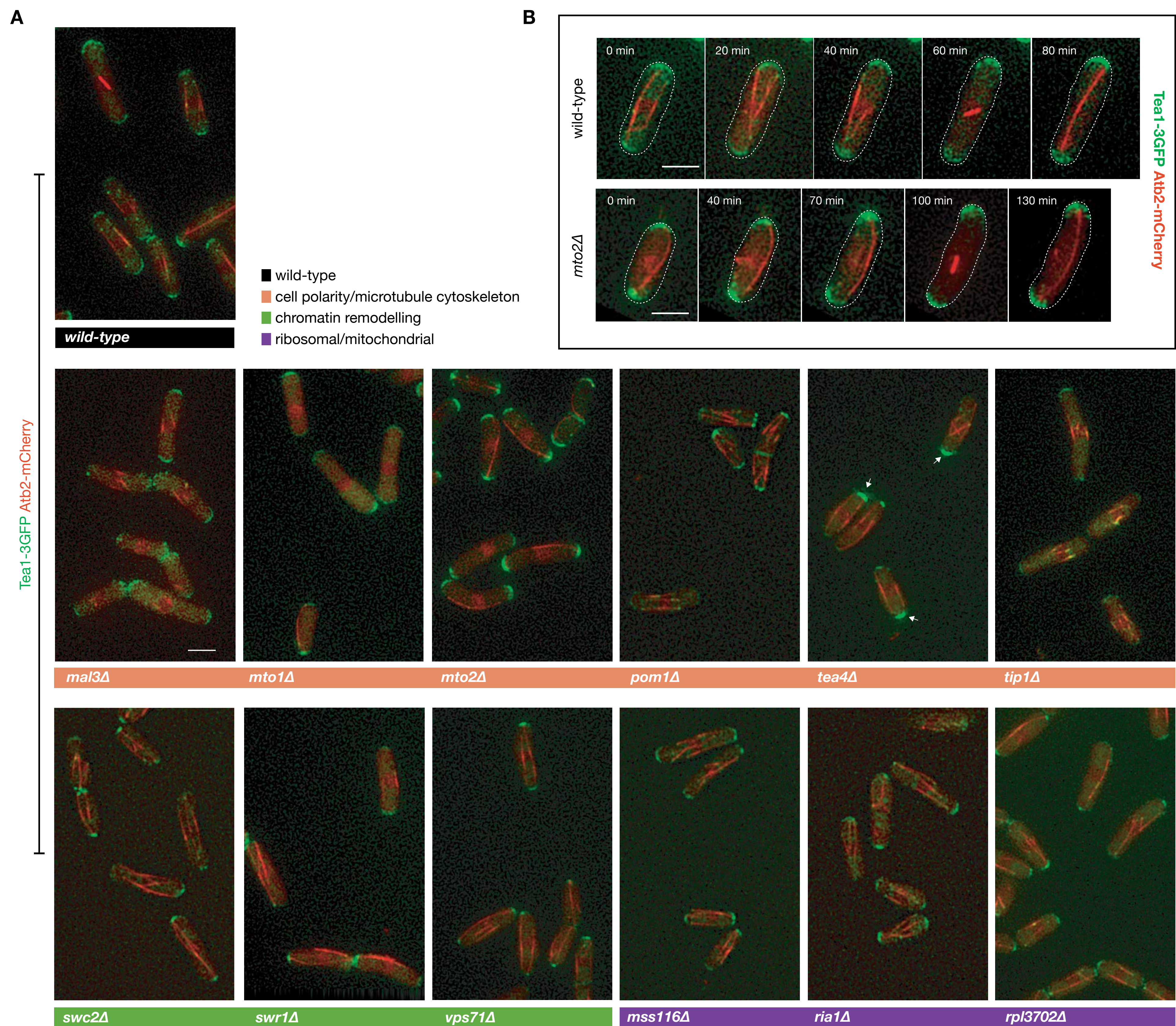

Abenza et al, Supplementary Material Figure S5

Supplement: Figure S5 — Distribution of the microtubule cytoskeleton and a polarity factor in curved mutants. A) Images of cells of 12 of the curved mutants and the wild-type that express Atb2-mCherry and Tea1-3GFP. The images were acquired via optical axis integration (OAI), which summed in a single frame all the information contained inside the cell (separation between top and bottom of the sample: 5 µm). The arrows in the tea4Δ image point abnormally high concentrations of Tea1-3GFP at the non growing end. B) Image sequence of an mto2Δ cell curving after misplacing Tea1-3GFP at the tips through its aberrant unique Atb2-mCherry microtubule bundle. Bars, 5 µm. (PDF) [file pone.0106959.s005.pdf]
