# Supplementary material for: Dynamics of Cell Shape Inheritance in Fission Yeast
Source: PLoS One. 2014 Sep 11;9(9):e106959. doi: 10.1371/journal.pone.0106959 (PMC4161360; doi:10.1371/journal.pone.0106959)
Supplement: Figure S4 — Examples of changes in cell morphology during growth. A tea1Δ monopolar straight cell and a tip1Δ monopolar curved cell were imaged during their whole cycle of growth. Images showing their morphology at the beginning of the movie and after 30, 60 and 90 minutes are displayed on the top. The panels in the middle represent the evolution of the cell outline curvature during growth. For both cells the peak on the left corresponds to the non growing end, whereas the peak on the right represents the evolving growing end. A negative curvature indicates a concavity in the cell outline. Superpositions of the successive time frames are displayed at the bottom to show the contrast between the maintenance of inherited structures and the constant morphological evolution of the growing tips. Bars, 5 µm. (PDF) [file pone.0106959.s004.pdf]

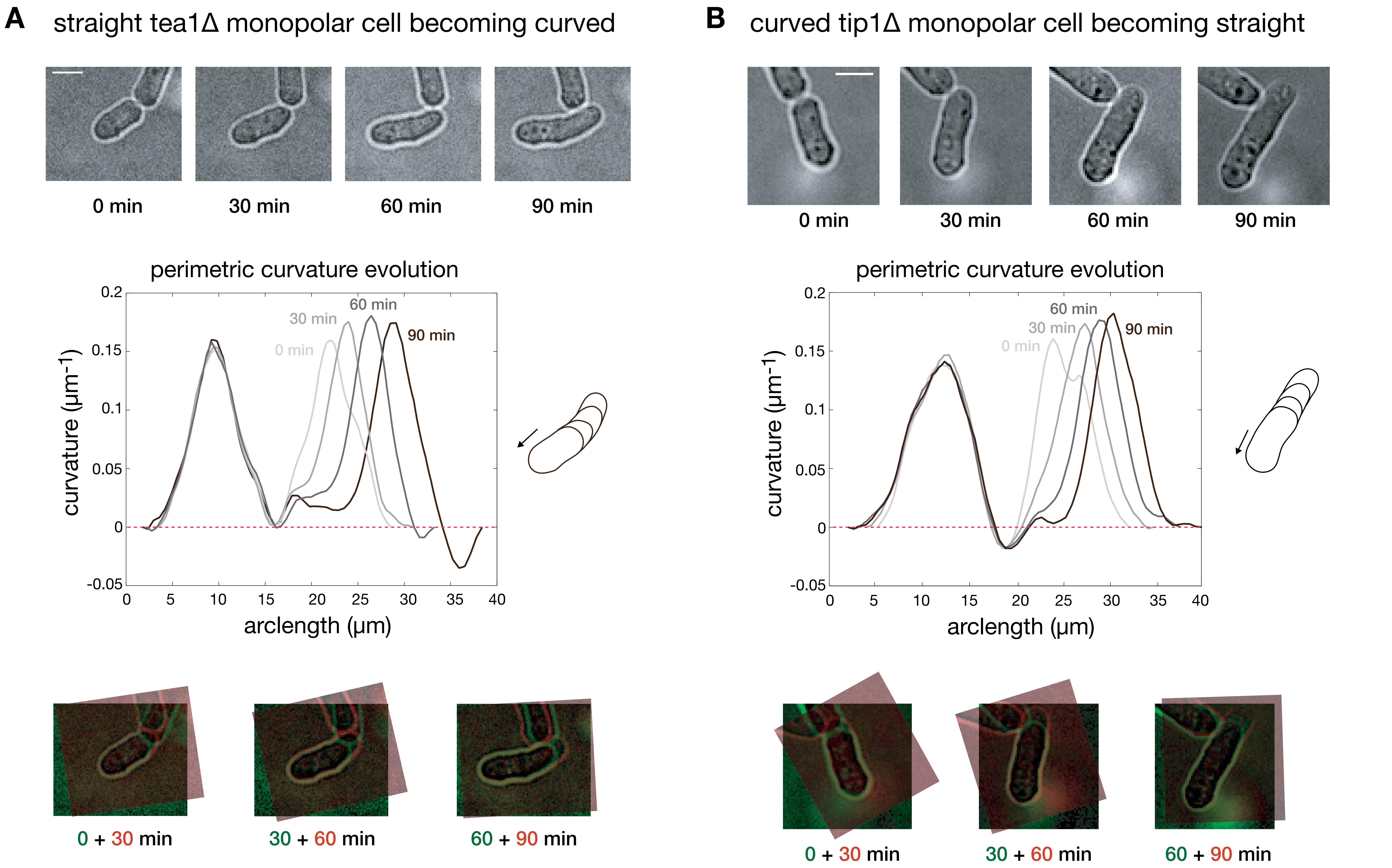

Abenza et al, Supplementary Material Figure S4
