# Supplementary material for: Dynamics of Cell Shape Inheritance in Fission Yeast
Source: PLoS One. 2014 Sep 11;9(9):e106959. doi: 10.1371/journal.pone.0106959 (PMC4161360; doi:10.1371/journal.pone.0106959)
Supplement: Figure S6 — Distribution of the actin cables and patches in curved mutants. Images of cells of 12 of the curved mutants and the wild-type that express GFP-lifeact. The images are maximal intensity projections of Z-stacks (separation between the 27 Z-planes: 0.2 µm). (PDF) [file pone.0106959.s006.pdf]

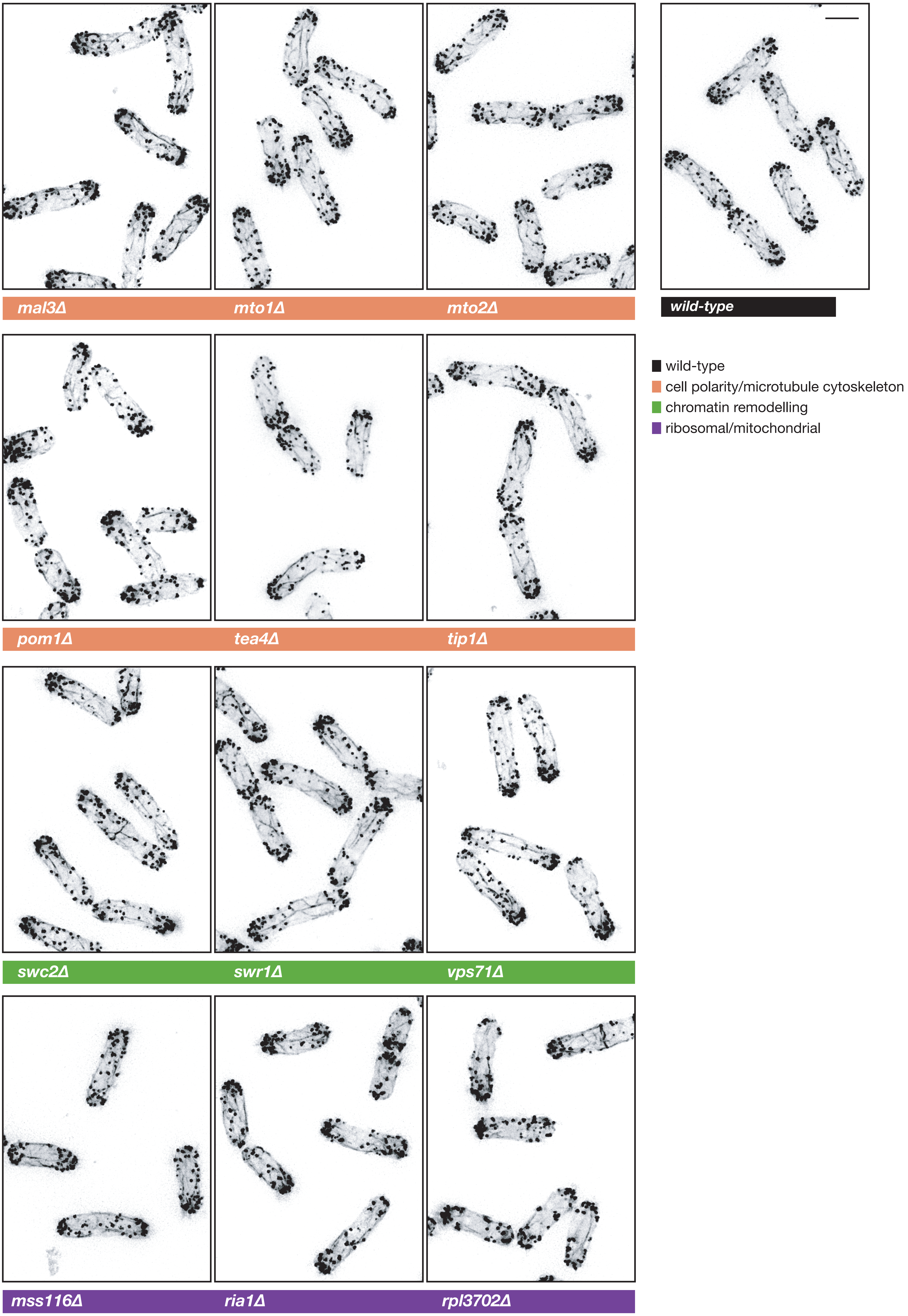

Abenza et al, Supplementary Material Figure S6
