# Supplementary material for: Dynamics of Cell Shape Inheritance in Fission Yeast
Source: PLoS One. 2014 Sep 11;9(9):e106959. doi: 10.1371/journal.pone.0106959 (PMC4161360; doi:10.1371/journal.pone.0106959)
Supplement: Table S1 — Strains used in this study. (DOCX) [file pone.0106959.s007.docx]

**Table S1: Strains used in this study.**

| Strain # | Genotype | Reference |
| --- | --- | --- |
|  |  |  |
| Deletion collection | *X∆::kan ade leu1-32 ura4-∆18* h+ (“x” refers to any non-essential gene which deletion generates a ‘curved/bent’ phenotype in *S. pombe*). | Bioneer [S[1](#_ENREF_1)] |
| JFA175 | h+ *ade6-M210 leu1-32 ura4-∆18 mat1_m-cyh smt-0 rpl42::cyhR (sP56Q) tea2∆::kan tip1∆::hph* | This study |
| JFA176 | h+ *ade6-M210 leu1-32 ura4-∆18 mat1_m-cyhS, smt-0 rpl42::cyhR (sP56Q) tea1∆::kan tip1∆::hph* | This study |
| JFA177 | h+ *ade6-M210 leu1-32 ura4-∆18 mat1_m-cyhS, smt-0 rpl42::cyhR (sP56Q) vps71∆::kan tea4∆::hph* | This study |
| JFA178 | h+ *ade6-M210 leu1-32 ura4-∆18 mat1_m-cyhS, smt-0 rpl42::cyhR (sP56Q) tea1∆::kan tea4∆::hph* | This study |
| JFA179 | h+ *ade6-M210 leu1-32 ura4-∆18 mat1_m-cyhS, smt-0 rpl42::cyhR (sP56Q) tea1∆::kan tea2∆::hph* | This study |
| JFA182 | h+ *ade6-M210 leu1-32 ura4-∆18 mat1_m-cyhS, smt-0 rpl42::cyhR (sP56Q) tea2∆::kan tea4∆::hph* | This study |
| JFA183 | h+ *ade6-M210 leu1-32 ura4-∆18 mat1_m-cyhS, smt-0 rpl42::cyhR (sP56Q) vps71∆::kan tip1∆::hph* | This study |
| JFA184 | h+ *ade6-M210 leu1-32 ura4-∆18 mat1_m-cyhS, smt-0 rpl42::cyhR (sP56Q) tea1∆::kan vps71∆::hph* | This study |
| JFA185 | h+ *ade6-M210 leu1-32 ura4-∆18 mat1_m-cyhS, smt-0 rpl42::cyhR (sP56Q) tea2∆::kan vps71∆::hph* | This study |
| JFA186 | h+ *ade6-M210 leu1-32 ura4-∆18 mat1_m-cyhS, smt-0 rpl42::cyhR (sP56Q) tip1∆::kan tea4∆::hph* | This study |
| JFA208 | *mto1∆::kan tea1-3GFP-LK-nat(MH222) mCherry-atb2-hph ura4-∆18? leu1-32? ade6-M210?* | This study |
| JFA209 | *mal3∆::kan tea1-3GFP-LK-nat(MH222) mCherry-atb2-hph ura4-∆18? leu1-32? ade6-M210?* | This study |
| JFA210 | *tip1∆::kan tea1-3GFP-LK-nat(MH222) mCherry-atb2-hph ura4-∆18? leu1-32? ade6-M210?* | This study |
| JFA211 | *swc2∆::kan tea1-3GFP-LK-nat(MH222) mCherry-atb2-hph ura4-∆18? leu1-32? ade6-M210?* | This study |
| JFA212 | *vps71∆::kan tea1-3GFP-LK-nat(MH222) mCherry-atb2-hph ura4-∆18? leu1-32? ade6-M210?* | This study |
| JFA213 | *pom1∆::kan tea1-3GFP-LK-nat(MH222) mCherry-atb2-hph ura4-∆18? leu1-32? ade6-M210?* | This study |
| JFA214 | *mss116∆::kan tea1-3GFP-LK-nat(MH222) mCherry-atb2-hph ura4-∆18? leu1-32? ade6-M210?* | This study |
| JFA215 | *rpl3702∆::kan tea1-3GFP-LK-nat(MH222) mCherry-atb2-hph ura4-∆18? leu1-32? ade6-M210?* | This study |
| JFA216 | *tea4∆::kan tea1-3GFP-LK-nat(MH222) mCherry-atb2-hph ura4-∆18? leu1-32? ade6-M210?* | This study |
| JFA217 | *swr1∆::kan tea1-3GFP-LK-nat(MH222) mCherry-atb2-hph ura4-∆18? leu1-32? ade6-M210?* | This study |
| JFA220 | *ria1∆::kan tea1-3GFP-LK-nat(MH222) mCherry-atb2-hph ura4-∆18? leu1-32? ade6-M210?* | This study |
| JFA227 | *mto2∆::hph tea1-3GFP-LK-nat(MH222) mCherry-atb2-hph ura4-∆18? leu1-32? ade6-M210?* | This study |
| JFA256 | *swc2::kan co2::Pdis2-GFP-linker-lifeact-Tact-nat his7? leu1 ura4 ade6-M216* | This study |
| JFA257 | *swr1::kan co2::Pdis2-GFP-linker-lifeact-Tact-nat his7? leu1 ura4 ade6-M216* | This study |
| JFA261 | *mal3::kan co2::Pdis2-GFP-linker-lifeact-Tact-nat his7? leu1 ura4 ade6-M216* | This study |
| JFA262 | *rpl3702::kan co2::Pdis2-GFP-linker-lifeact-Tact-nat his7? leu1 ura4 ade6-M216* | This study |
| JFA263 | *pom1::kan co2::Pdis2-GFP-linker-lifeact-Tact-nat his7? leu1 ura4 ade6-M216* | This study |
| JFA264 | *vps71::kan co2::Pdis2-GFP-linker-lifeact-Tact-nat his7? leu1 ura4 ade6-M216* | This study |
| JFA265 | *mto1::kan co2::Pdis2-GFP-linker-lifeact-Tact-nat his7? leu1 ura4 ade6-M216* | This study |
| JFA266 | *mss116::kan co2::Pdis2-GFP-linker-lifeact-Tact-nat his7? leu1 ura4 ade6-M216* | This study |
| JFA267 | *tea4::kan co2::Pdis2-GFP-linker-lifeact-Tact-nat his7? leu1 ura4 ade6-M216* | This study |
| JFA268 | *mto2::hph co2::Pdis2-GFP-linker-lifeact-Tact-nat his7? leu1 ura4 ade6-M216* | This study |
| JFA269 | *ria1::kan co2::Pdis2-GFP-linker-lifeact-Tact-nat his7? leu1 ura4 ade6-M216* | This study |
| JFA271 | *tip1::hph co2::Pdis2-GFP-linker-lifeact-Tact-nat his7? leu1 ura4 ade6-M216* | This study |
| RCS446 | *co2::Pdis2-GFP-linker-lifeact-Tact-nat his7 leu1 ura4 ade6-M216* | M. Sato |
| MGP10-h | *tip1∆::hph ade-M210 leu1-32 ura4-∆18* h+ | M. Geymonat |
| MGP12-h | *tea4∆::hph ade-M210 leu1-32 ura4-∆18* h+ | M. Geymonat |
| MGP14 PEM | h- *ade6-M210 leu1-32 ura4-∆18 mat1_m-cyhS, smt-0 rpl42::cyhR (sP56Q) vps71∆::kan* | M. Geymonat* |
| MGP14-h | *vps71∆::hph ade-M210 leu1-32 ura4-∆18* h+ | M. Geymonat |
| MGP14-h | *vps71∆::hph ade-M210 leu1-32 ura4-∆18* h+ | M. Geymonat |
| MGP15 PEM | *h- ade6-M210 leu1-32 ura4-∆18 mat1_m-cyhS, smt-0 rpl42::cyhR (sP56Q) tea1∆::kan* | M. Geymonat* |
| MGP4 PEM | h- *ade6-M210 leu1-32 ura4-∆18 mat1_m-cyhS, smt-0 rpl42::cyhR (sP56Q) tea2∆::kan* | M. Geymonat* |
| MGP4-h | *tea2∆::hph ade-M210 leu1-32 ura4-∆18* h+ | M. Geymonat |
| PN557 | *ade-M216 leu1-32 ura4-∆18* h- | P. Nurse |
| RCS432 | *tea1-3GFP-LK-nat(MH222) mCherry-atb2-hph* | J. Dodgson |
| RCS755 | h- *ade6-M210 leu1-32 ura4-∆18 mat1_m-cyhS, smt-0 rpl42::cyhR (sP56Q) tip1∆::kan* | J. Dodgson* |
| RCS883 | *swc2∆::hph ade-M210 leu1-32 ura4-∆18* h- | This study |
| RCS895 | *swr1∆::hph ade-M210 leu1-32 ura4-∆18* h- | This study |
| RCS900 | *swc2∆::hph swr1∆::kan ade-M210 leu1-32 ura4-∆18* | This study |
| RCS904 | *swc2∆::hph tip1∆::kan ade-M210 leu1-32 ura4-∆18* | This study |
| RCS905 | *swc2∆::hph vps71∆::kan ade-M210 leu1-32 ura4-∆18* | This study |
| RCS907 | *swc2∆::hph tea1∆::kan ade-M210 leu1-32 ura4-∆18* | This study |
| RCS920 | *swr1∆::hph tip1∆::kan ade-M210 leu1-32 ura4-∆18* | This study |
| RCS934 | *swr1∆::hph tea4∆::kan ade-M210 leu1-32 ura4-∆18* h+ | This study |
| RCS935 | *swr1∆::hph tea2∆::kan ade-M210 leu1-32 ura4-∆18* | This study |
| RCS936 | *swr1∆::hph tea1∆::kan ade-M210 leu1-32 ura4-∆18* | This study |
| RCS937 | *swr1∆::hph vps71∆::kan ade-M210 leu1-32 ura4-∆18* | This study |
| RCS938 | *swc2∆::hph tea2∆::kan ade-M210 leu1-32 ura4-∆18* | This study |
| RCS939 | *swc2∆::hph tea4∆::kan ade-M210 leu1-32 ura4-∆18* | This study |

* Derived from P392: h- *ade6-M210 leu1-32 ura4-∆18 mat1_m-cyhS smt-0 rpl42::cyhR (sP56Q) vps71∆::kan* [S[2](#_ENREF_2)]

**Bibliography**

S1. Kim, D.U., Hayles, J., Kim, D., Wood, V., Park, H.O., Won, M., Yoo, H.S., Duhig, T., Nam, M., Palmer, G., et al. (2010). Analysis of a genome-wide set of gene deletions in the fission yeast *Schizosaccharomyces pombe*. Nature biotechnology 28, 617-623.

S2. Roguev, A., Wiren, M., Weissman, J.S., and Krogan, N.J. (2007). High-throughput genetic interaction mapping in the fission yeast *Schizosaccharomyces pombe*. Nature methods 4, 861-866.
