# Supplementary material for: Dynamics of Cell Shape Inheritance in Fission Yeast
Source: PLoS One. 2014 Sep 11;9(9):e106959. doi: 10.1371/journal.pone.0106959 (PMC4161360; doi:10.1371/journal.pone.0106959)
Supplement: Table S3 — Detailed information of a specific tip1Δ lineage across four generations. The column on the left shows the cells’ names. Each cell, except the first one (*1) contains two sets of data, represented in two rows. The first one (from the top to the bottom of the table) corresponds to the measurements taken after the mother’s cell division, whereas the second one shows the measurements taken before the cell started septating. These data belong to the lineage shown in Fig. 1C and Movie S1. (DOCX) [file pone.0106959.s009.docx]

| cellID | time (min) | length (µm) | radius (µm) | inverted radius (µm^-1^) |
| --- | --- | --- | --- | --- |
|  |  |  |  |  |
| *1 | 20 | 20.9910 | 21.8033 | 0.0459 |
| *1.1 | 60 | 11.6090 | 16.2075 | 0.0617 |
| *1.2 | 60 | 11.2054 | 76.5711 | 0.0131 |
| *1.1 | 170 | 19.2519 | 16.7302 | 0.0598 |
| *1.2 | 120 | 14.4092 | 102.4389 | 0.0098 |
| *1.1.1 | 190 | 9.4216 | 115.4866 | 0.0694 |
| *1.1.2 | 190 | 11.7645 | 43.1034 | 0.0232 |
| *1.2.1 | 150 | 8.5704 | 52.1971 | 0.0192 |
| *1.2.2 | 150 | 8.4732 | 51.6757 | 0.0194 |
| *1.1.1 | 290 | 16.9985 | 33.7430 | 0.0296 |
| *1.1.2 | 290 | 18.9663 | 28.7815 | 0.0347 |
| *1.2.1 | 270 | 17.5983 | 29.8903 | 0.0335 |
| *1.2.2 | 280 | 17.2957 | 92.8580 | 0.0108 |
| *1.1.1.1 | 320 | 9.5627 | 23.9114 | 0.0418 |
| *1.1.1.2 | 320 | 9.5444 | 30.2976 | 0.0330 |
| *1.1.2.1 | 310 | 9.5497 | 23.1673 | 0.0432 |
| *1.1.2.2 | 310 | 11.6361 | 113.6903 | 0.0088 |
| *1.2.1.1 | 290 | 9.3666 | 28.5643 | 0.0350 |
| *1.2.1.2 | 290 | 10.3650 | 13.4665 | 0.0743 |
| *1.2.2.1 | 300 | 9.1452 | 26.6478 | 0.0375 |
| *1.2.2.2 | 300 | 9.9313 | 22.6345 | 0.0442 |
| *1.1.1.1 | 390 | 16.2311 | 28.2141 | 0.0354 |
| *1.1.1.2 | 390 | 15.8308 | 82.4075 | 0.0121 |
| *1.1.2.1 | 400 | 17.6427 | 16.6718 | 0.0600 |
| *1.1.2.2 | 370 | 17.9173 | 75.7994 | 0.0132 |
| *1.2.1.1 | 370 | 15.8946 | 21.7104 | 0.0461 |
| *1.2.1.2 | 360 | 15.5482 | 17.0239 | 0.0587 |
| *1.2.2.1 | 380 | 15.5940 | 274.1644 | 0.0036 |
| *1.2.2.2 | 370 | 17.1522 | 16.9010 | 0.0592 |
|  |  |  |  |  |

**Table S3.**  Detailed information of a specific *tip1∆* lineage across four generations. The column on the left shows the cells' names. Each cell, except the first one (*1) contains two sets of data, represented in two rows. The first one (from the top to the bottom of the table) corresponds to the measurements taken after the mother's cell division, whereas the second one shows the measurements taken before the cell started septating. These data belong to the lineage shown in Fig. 1C and Supplementary Material Movie S1.
