# Supplementary material for: Dynamics of Cell Shape Inheritance in Fission Yeast
Source: PLoS One. 2014 Sep 11;9(9):e106959. doi: 10.1371/journal.pone.0106959 (PMC4161360; doi:10.1371/journal.pone.0106959)
Supplement: Table S4 — Overall penetrance of the curved phenotype in each of the strains listed. The number of cells measured varied depending on the mutant, from 112 to 497. Thus, cell shape is modulated by multiple and complex factors - cell wall inheritance, cell shape changes at division, active growth pattern changes, and likely many others -, which together give rise to the overall cell shape inheritance rules specific to each genotype. (DOCX) [file pone.0106959.s010.docx]

| Deletion(s) | Overall penetrance |
| --- | --- |
|  |  |
| *swc2∆* | 32.55% |
| *swr1∆* | 28.80% |
| *vps71∆* | 30.21% |
| *tea2∆* | 40.24% |
| *tea4∆* | 37.33% |
| *tea1∆* | 41.67% |
| *tip1∆* | 36.80% |
| *swr1∆ vps71∆* | 31.30% |
| *swr1∆ tip1∆* | 38.76% |
| *swr1∆ tea4∆* | 52.76% |
| *swr1∆ tea2∆* | 48.05% |
| *swr1∆ tea1∆* | 61.19% |
| *swc2∆ vps71∆* | 28.11% |
| *swc2∆ tip1∆* | 48.98% |
| *swc2∆ tea1∆* | 54.82% |
| *swc2∆ tea4∆* | 64.00% |
| *swc2∆ swr1∆* | 21.09% |
| *vps71∆ tea4∆* | 56.29% |
| *tip1∆ tea4∆* | 45.64% |
| *tip1∆ vps71∆* | 39.86% |
| *tip1∆ tea2∆* | 39.57% |
| *tea2∆ vps71∆* | 48.03% |
| *tea2∆ tea4∆* | 44.38% |
| *tea1∆ vps71∆* | 63.69% |
| *tea1∆ tip1∆* | 36.98% |
| *tea1∆ tea4∆* | 43.65% |
| *tea1∆ tea2∆* | 38.94% |
| *swc2∆ tea2∆* | 56.49% |
|  |  |

**Table S4.** Overall penetrance of the curved phenotype in each of the strains listed. The number of cells measured varied depending on the mutant, from 112 to 497.
